# Biocompatible Chitosan Oligosaccharide Modified Gold Nanorods as Highly Effective Photothermal Agents for Ablation of Breast Cancer Cells

**DOI:** 10.3390/polym10030232

**Published:** 2018-02-26

**Authors:** Panchanathan Manivasagan, Subramaniyan Bharathiraja, Madhappan Santha Moorthy, Sudip Mondal, Thanh Phuoc Nguyen, Hyehyun Kim, Thi Tuong Vy Phan, Kang Dae Lee, Junghwan Oh

**Affiliations:** 1 Marine-Integrated Bionics Research Center, Pukyong National University, Busan 48513, Korea; manimaribtech@gmail.com (P.M.); sbrbtc@gmail.com (S.B.); santham83@gmail.com (M.S.M.); mailsudipmondal@gmail.com (S.M.); hyki7732@colorado.edu (H.K.); phanvy120690@gmail.com (T.T.V.P.); 2 Department of Biomedical Engineering and Center for Marine-Integrated Biotechnology (BK21 Plus), Pukyong National University, Busan 48513, Korea; ntphuoc2000@gmail.com; 3 Department of Otolaryngology—Head and Neck Surgery, Kosin University Gospel Hospital, Kosin University College of Medicine, 262 Gamcheon-ro, Seo-Gu, Busan 602-702, Korea; kdlee59@gmail.com

**Keywords:** gold nanorods, chitosan oligosaccharide, near-infrared, photothermal therapy

## Abstract

Photothermal therapy (PTT) using biocompatible nanomaterials have recently attracted much attention as a novel candidate technique for cancer therapy. In this work we report the performance of newly synthesized multidentate chitosan oligosaccharide modified gold nanorods (AuNRs-LA-COS) as novel agents for PTT of cancer cells due to their excellent biocompatibility, photothermal stability, and high absorption in the near-infrared (NIR) region. The AuNRs-LA-COS exhibit a strong NIR absorption peak at 838 nm with a mean length of 26 ± 3.1 nm and diameter of 6.8 ± 1.7 nm, respectively. The temperature of AuNRs-LA-COS rapidly reached 52.6 °C for 5 min of NIR laser irradiation at 2 W/cm^2^. The AuNRs-LA-COS had very low cytotoxicity and exhibited high efficiency for the ablation of breast cancer cells in vitro. The tumor-bearing mice were completely ablated without tumor recurrence after photothermal treatment with AuNRs-LA-COS (25 µg/mL) under laser irradiation. In summary, this study demonstrated that AuNRs-LA-COS with laser irradiation as novel agents pave an alternative way for breast cancer therapy and hold great promise for clinical trials in the near future.

## 1. Introduction

Photothermal therapy (PTT) has recently attracted considerable attention as a promising alternative for effective cancer therapy [1]. PTT is a minimally invasive and laser-based technique to especially “burn” cancer cells in the presence of light absorbing agents, while minimizing damage to the surrounding healthy tissue [2,3,4,5]. PTT also enables rapid recovery than traditional clinical therapeutic methods such as chemotherapy, radiotherapy, and surgical management [6,7]. In PTT, photon energy is converted into cytotoxic heat to destroy cancer cells, by causing irreversible tissue damage through thermal denaturation of proteins and tissue coagulation [3,8]. Nanomaterial-mediated PTT using NIR is a highly efficient alternative to cancer therapy [9]. NIR light absorption materials in the range of 700 to 1000 nm is the optimum condition for PTT because of the high transparency window of tissue in this range [10,11,12].

Gold nanorods (AuNRs) have recently attracted considerable attention as promising nanomaterials for their photothermal responsive properties such as strong absorption in the NIR region, biocompatibility, and photostability [13,14,15]. AuNRs were proven to be the most suitable for PTT because of their strong scattering and absorption in the NIR region, including a better heat generation rate than other nanoparticles [7,16]. AuNRs are synthesized via the seed-mediated route and are capped by cetyltrimethyl ammonium bromide (CTAB) bilayers [17]. AuNRs have limited clinical use due to cytotoxicity (toxicity to cells) caused by the residual CTAB. To reduce such cytotoxicity, the marine biopolymer chitosan oligosaccharide (COS) is widely used for surface modification of AuNRs [18,19,20,21,22,23].

Marine biopolymer-based nanomaterials have therefore emerged as potential candidates for therapeutic applications [24]. Chitosan is a natural biopolymer and an attractive biomaterial for therapeutic applications because of their excellent biocompatibility, biodegradability, and nontoxicity. However, the major drawback of chitosan is its poor solubility at neutral pH [25,26]. To overcome these inherent drawbacks, COS was developed to serve as a novel candidate for biomedical applications. COS is a de-polymerization product of chitosan and are cationic, with low molecular weight, water solubility, abundance, biocompatibility, biodegradability, and nontoxicity in nature [27,28]. COS has reactive hydroxyl and amino groups, for each polymer subunit that permits ionic cross linking [29]. Multidentate ligands or dithiol ligands, including lipoic acid (LA), are widely used to bind with high affinity to AuNRs [30,31]. The LA was conjugated to COS using carbodiimide chemistry, which produced LA-COS. The LA-COS was employed to replace cetryltrimethylammonium bromide (CTAB) on the surface of the AuNRs synthesized by the seed-mediated route [32,33,34]. By combining characteristics of these materials AuNRs-LA-COS have gained increased attention for PTT due to their excellent biocompatibility, photothermal stability, and high absorption in the NIR region. In this work, we developed AuNRs-LA-COS as novel agents for photothermal therapy.

## 2. Experimental Section

### 2.1. Synthesis and Surface Modification of Gold Nanorods

AuNRs were synthesized using the seed-mediated technique according to the previous literature with some modifications [32,33,34]. The detailed synthesis procedure was previously reported by our group [35]. The AuNRs-LA-COS were synthesized according to previous literature with slight modifications [23,36]. Briefly, 2 g of a 0.4 mmol of COS, 0.576 g of a 3 mmol of 1-(3-dimethylaminopropyl)-3-ethylcarbodiimide hydrochloride (EDC·HCl), and 1.302 g of a 6 mmol *N*-hydroxysulfosuccinimide sodium (sulfo-NHS) were dissolved in 50 mL of distilled water while stirring at room temperature. 0.494 g of a 2.4 mmol of LA was dissolved in hot ethanol at 60 °C. After the solution completely dissolved, LA solution was added in COS aqueous solution and further stirred at 24 °C. The LA-COS aqueous solution was purified by dialysis using a membrane (MWCO: 3.5 kDa) for 3 days, followed by lyophilization. LA-COS (0.5 mg/mL) was dissolved in 10 mL of distilled water and the LA-COS solution was added to 20 mL of purified AuNRs while stirring at room temperature for 24 h. The modified AuNRs (AuNRs-LA-COS) were purified with four rounds of centrifugation at 12,000 rpm for 15 min, to remove excess COS and obtain AuNRs-LA-COS. The amino substitution degree of COS-LA conjugate was determined by 2,4,6-trinitrobenzene sulfonic acid (TNBS) method [37].

### 2.2. Stability Studies

The stability of colloidal AuNRs and AuNRs-LA-COS suspensions were carefully monitored up to six months by UV-Vis spectrophotometry. The stability of AuNRs-LA-COS was performed in various pH and NaCl concentrations. The AuNRs-LA-COS were dispersed in distilled water and pH adjusted (pH 2.0–13.0), and the AuNRs-LA-COS dispersion was monitored at various concentrations of NaCl (0.1 to 0.5 M). To verify the stability, the AuNR-LA-COS were dispersed in distilled water (DW), phosphate-buffered saline (PBS), and a cell culture medium supplemented with 10% fetal bovine serum (FBS) (DMEM without phenol red). The aqueous solutions were maintained at 25 °C, monitored for 7 days, and UV-Vis spectra of aqueous solutions taken periodically.

### 2.3. In Vitro Photothermal Heating Characterization

To measure the in vitro photothermal heating efficiency of AuNRs-LA-COS, aqueous solutions of different concentrations (15, 20, and 25 µg/mL) were introduced in a 35 mm cell culture plate (1 mL) and irradiated with 808 nm NIR laser (Changchun New Industries Optoelectronics Technology, Changchun, China) at different power densities (0.5, 1.0, 1.5, and 2.0 W/cm^2^) for 5 min, respectively. The temperatures of each of the solutions were measured every 1 s by a digital thermometer, with a thermocouple probe and a FLIR i5 infrared (IR) camera (Flir Systems Inc., Portland, OR, USA). DW was chosen as the control.

To investigate the photostability of AuNRs-LA-COS (25 µg/mL), the solutions were irradiated with an 808 nm NIR laser at 2 W/cm^2^ for 5 min and then cooled naturally to room temperature for 15 min without 808 nm NIR laser irradiation. This procedure was repeated for 6 cycles and the irradiated samples measured by UV-Vis-NIR spectroscopy.

### 2.4. Cell Viability Assay

In vitro cytotoxicity of AuNRs-LA-COS was evaluated by MTT cell viability assay of human breast cancer cells (MDA-MB-231). Cells were seeded into a 96-well microplate at a density of 1 × 10^4^ cells/well in DMEM medium and incubated overnight for cell adhesion. The cell culture media was replaced with fresh medium containing varying concentrations of the AuNRs-LA-COS (10 to 100 µg/mL) for 24 and 48 h at 37 °C under 5% CO_2_. Thereafter, 100 µL of MTT containing DMEM medium (0.5 mg/mL) was added to each well and cultured for 4 h at 37 °C. After removal of the medium, the purple formazan was mixed with DMSO for 15 min. The absorbance of the solution was measured at 540 nm using a microplate reader (BioTek, PowerWave XS2, Winooski, VT, USA).

### 2.5. In Vitro Photothermal Ablation of MDA-MB-231 Cells

MDA-MB-231 cells were seeded into 96-well plates at a density of 1 × 10^4^ cells/well and incubated for 24 h at 37 °C. Afterwards, the cells were treated with different concentrations of AuNRs-LA-COS (5, 10, 15, 20, and 25 µg/mL) for 4 h and irradiated with or without 808 nm NIR laser at 2.0 W/cm^2^ for 5 min. Thereafter, cells were further incubated for 2 h to measure cytotoxicity using the MTT assay.

MDA-MB-231 cells were seeded into 96-well plates with 200 µL fresh DMEM medium at a density of 1 × 10^4^ cells/well and incubated for 24 h at 37 °C. After incubation, cells were randomly assigned into four groups: group I, control cells; group II, control cells + 808 nm laser; group III, AuNRs-LA-COS; and group IV, AuNRs-LA-COS + 808 nm laser. The group I had no treatment and the group II was irradiated for 5 min the different power densities of 0.5, 1.0, 1.5, and 2.0 W/cm^2^, respectively. The group III and IV were treated with 25 µg/mL AuNRs-LA-COS for 4 h and irradiated without or with 808 nm NIR laser at different power densities of 0.5, 1.0, 1.5, and 2.0 W/cm^2^, respectively. After irradiation treatment, cells were incubated for 2 h and the cell viability measured by MTT assay. In parallel, the morphology of cells was observed in MDA-MB-231 treated without or with 25 µg/mL AuNRs-LA-COS and irradiated without or with 808 nm NIR laser at 2 W/cm^2^ for 5 min. The cells were observed using an inverted phase contrast microscope (Leica Microsystems GmbH, Wetzlar, Germany). For trypan blue staining, the group of cells were stained with 0.4% trypan blue solution and observed under an inverted phase contrast microscope (Leica Microsystems GmbH) to differentiate dead cells.

For fluorescent microscope observations, MDA-MB-231 cells were seeded into 6-well plates at a density of 2 × 10^5^ cells/well and incubated for 24 h. After incubation, the cells were treated without or with 25 µg/mL AuNRs-LA-COS and irradiated without or with 808 nm NIR laser at 2 W/cm^2^ for 5 min. After incubating for another 2 h, all the cells were stained with acridine orange (AO) and propidium iodide (PI) for 30 min, which could stain the cells to distinguish the living cells (green) from the dead ones (red) and observed using an Leica DMI300B fluorescence microscope (Leica Microsystems GmbH).

For confocal laser scanning microscopy, MDA-MB-231 (2 × 10^5^ cells) cells were seeded in a 35 mm glass-bottomed cell culture dish in 2 mL of DMEM medium and cultured for 24 h. Afterward, the cells were treated without or with 25 µg/mL AuNRs-LA-COS and irradiated with 808 nm NIR laser at 2 W/cm^2^ for 5 min. The cells were cultured for an additional 2 h and the cells were washed thrice with PBS. The cells were stained with DAPI (4′,6-diamidine-2′-phenylindole dihydrochloride) and MitoTracker Red or DAPI and LysoTracker green for 30 min and then the cells were washed with PBS three times. Confocal laser scanning microscopic analysis of these cells were done on a Zeiss LSM 700 confocal microscope.

For flow cytometry, cells were seeded in 6-well plates at a density of 5 × 10^5^ cells/well in DMEM medium and incubated for 24 h at 37 °C. After incubation, the cells were treated with or without AuNRs-LA-COS (25 µg/mL) for 4 h and the cells were treated with or without 808 nm NIR laser at 2 W/cm^2^ for 5 min. All the cells were further incubated for 2 h, cells were harvested and analyzed with a flow cytometer (BD FACSVerse, San Jose, CA, USA).

### 2.6. In Vivo Photothermal Ablation of MDA-MB-231 Cells

Female BALB/c nude mice (19–21 g body weight) were obtained from Orient Bio Inc. (Gyeonggi-Do, Korea). All animal studies were approved according to the institutional animal care and use committee of Pukyong National University. MDA-MB-231 (2 × 10^5^) were injected subcutaneously into the left flank of animals. The mice were chosen for in vivo studies when their tumor sizes reached about 130 mm^3^.

The animals were randomly divided into four groups (*n* = 5): group I, PBS injection; group II, PBS + NIR laser; group III, AuNRs-LA-COS; and group IV, AuNRs-LA-COS + NIR laser. The group I and II mice were intratumorally injected with 100 µL of PBS with or without 808 nm NIR laser at 2 W/cm^2^ for 5 min. The group III mice were intratumorally injected with 100 µL of 3 mg/mL AuNRs-LA-COS. The group IV mice were intratumorally injected with 100 µL of 3 mg/mL AuNRs-LA-COS and continuously irradiated with 808 nm NIR laser at 2 W/cm^2^ for 5 min under anesthetic conditions. Before irradiation, a thermometer probe was inserted into the tumor. Simultaneously, temperature variations of the tumor sites were recorded by a FLIR i5 infrared (IR) camera. Mice body weights were measured using a scale-balance and the tumor lengths and widths measured with a digital vernier caliper every day. Tumor volume was calculated as follows (1):

(1) $$\mathrm{Tumor}\;\mathrm{volume}\;=\frac{\left(\mathrm{Tumor}\;\mathrm{length}\right)\times {\left(\mathrm{Tumor}\;\mathrm{width}\right)}^{2}}{2}\;$$

### 2.7. Histology Analysis

For hematoxylin and eosin (H&E) staining, major organs (heart, kidney, spleen, lung, and liver) and tumor tissues were harvested, fixed in 10% neutral buffered formalin, paraffin embedded, sectioned at 5 µm, stained with H&E, and examined using an inverted phase contrast microscope (Leica Microsystems GmbH).

## 3. Results and Discussion

### 3.1. Surface Modification of AuNRs

**Scheme 1:**
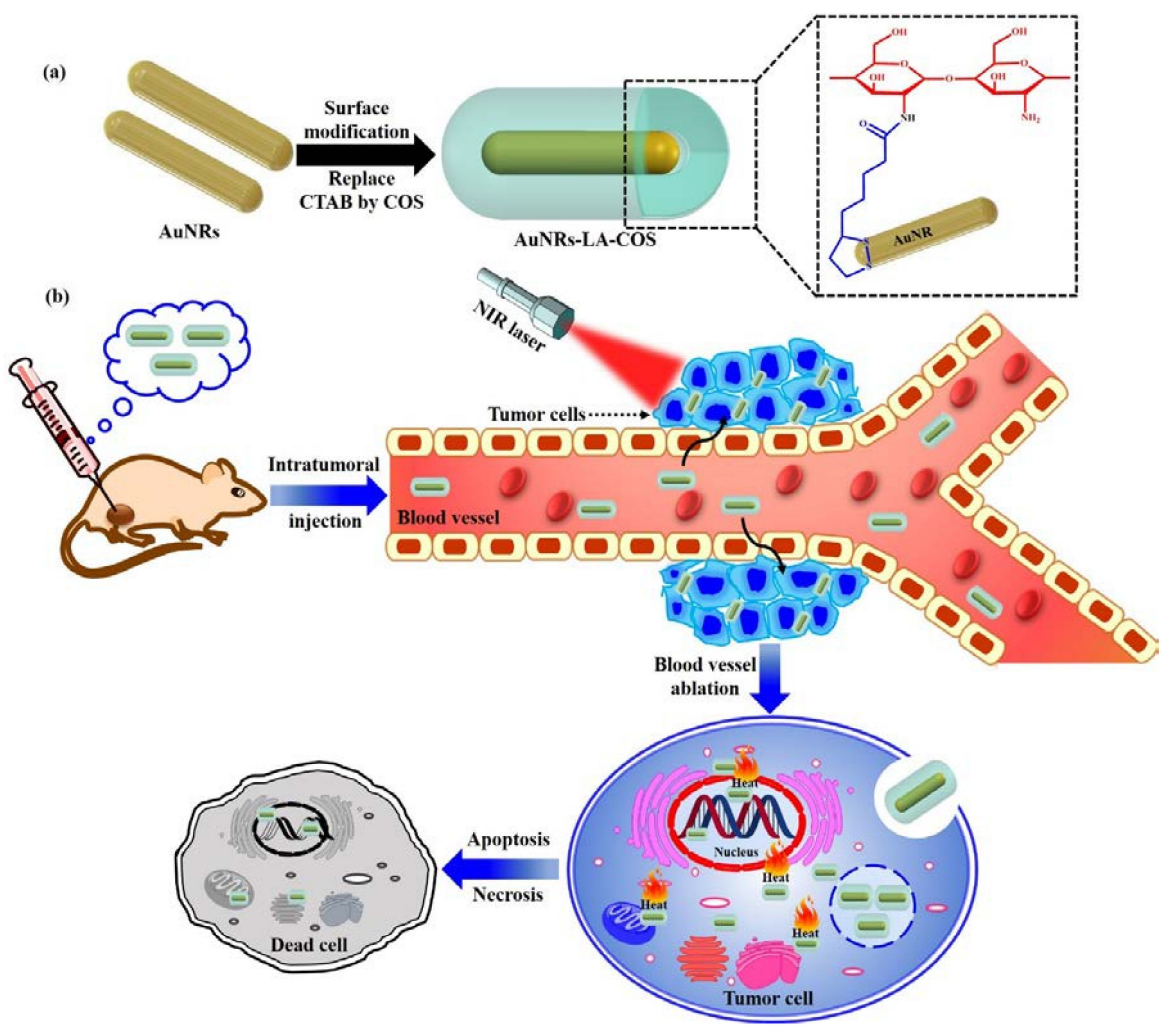
(**a**) Schematic illustration of AuNRs-LA-COS formation. (**b**) A scheme showing the possible mechanism of AuNRs-LA-COS for photothermal therapy.

COS is a depolymerization product of chitosan, which can be prepared from the deacetylation of chitin [25]. COS has reactive amino groups for each polymer subunit that permits ionic cross-linking [29]. The LA was conjugated to COS using carbodiimide chemistry, which produced LA-COS. The LA-COS was employed to replace CTAB on the surface of the AuNRs synthesized by the seed-mediated route. The preparation process of AuNRs-LA-COS for photothermal therapy is illustrated in Scheme 1a,b and Figure S1. The TNBS method was used to determine the degree of amino substitution of LA-COS. Our results indicated that the degree of amino substitution of prepared LA-COS was about 19.3% from the determination by TNBS method.

### 3.2. Characterization

**Figure 1:**
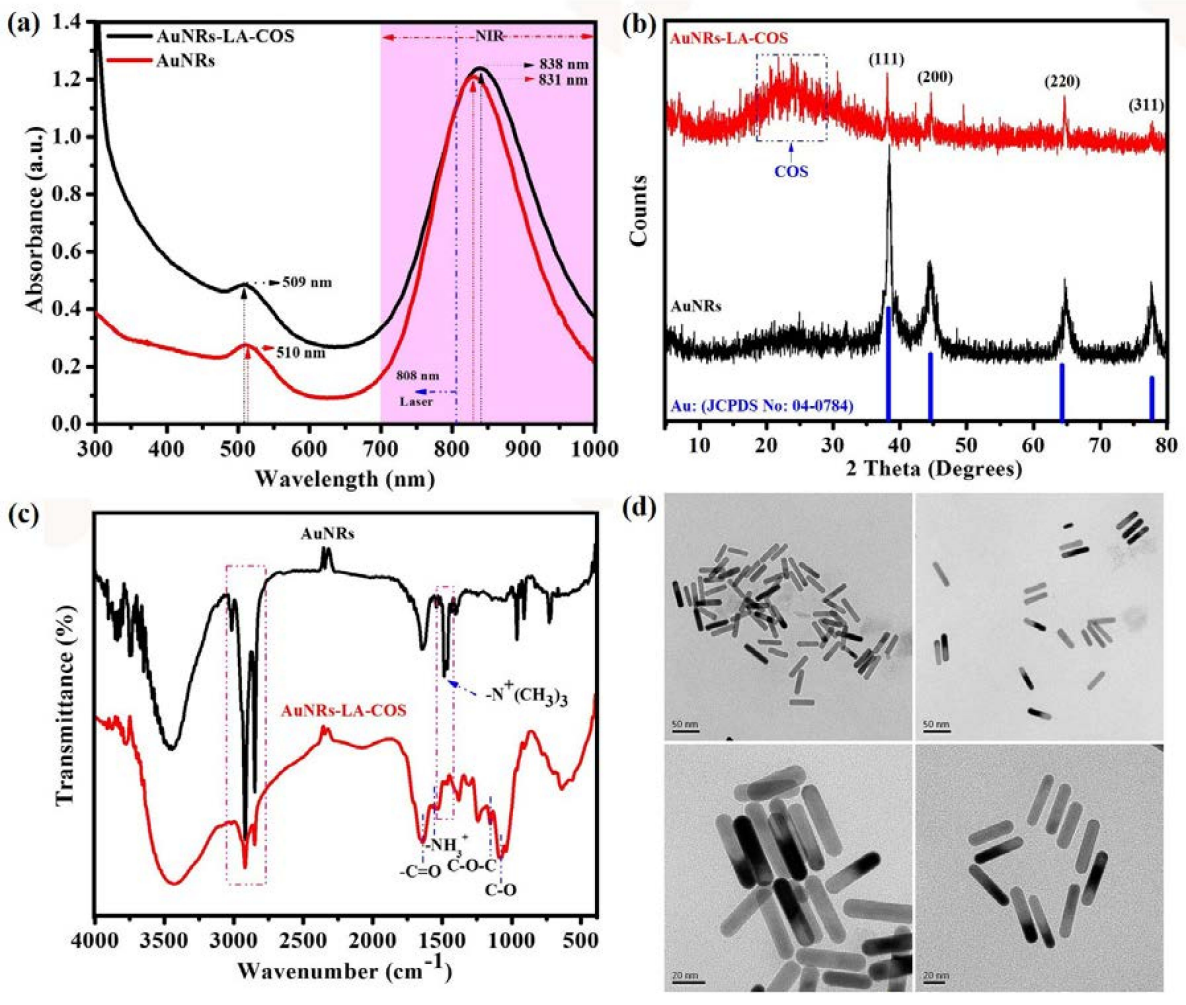
(**a**) UV-Vis-NIR absorbance spectra of AuNRs and AuNRs-LA-COS. (**b**) XRD patterns of AuNRs and AuNRs-LA-COS. (**c**) FTIR spectrum of AuNRs and AuNRs-LA-COS. (**d**) FETEM image of AuNRs-LA-COS.

The absorption spectra of the AuNRs and AuNRs-LA-COS were obtained by UV-Vis spectrophotometry (Figure 1a). AuNRs have a weak short transverse plasmon band at 510 nm and a strong long longitudinal surface plasmon resonance (SPR) band at 831 nm. After surface modification of AuNRs, a dramatic red-shift in the long longitudinal SPR band from ~831 nm to 838 nm was observed and this could be because of the change in the local refractive index around the AuNRs, which is sensitive to the changes on AuNRs surface. Similar results were reported to show that coating of polymers can induce the longitudinal SPR band shift from the AuNRs [38,39].

The X-ray diffraction (XRD) pattern was used to characterize the crystalline phase of AuNRs and AuNRs-LA-COS (Figure 1b). The XRD peaks from atomic lattices of the AuNRs were clearly seen that peak at 2*θ* = 38.13°, 44.73°, 64.93°, and 77.54°, assigned as (111), (200), (220), and (311) reflection lines, respectively. The XRD pattern of COS showed a major characteristic peak at 23.14°. The XRD pattern of AuNRs-LA-COS showed five characteristic peaks at 23.14°, 38.13°, 44.54°, 64.53° and 77.92°, which clearly demonstrated the characteristic peaks associated with COS and AuNRs. These peaks were consistent with the joint committee on powder diffraction standards (JCPDS 04-0784) and earlier reports [40,41]. The Fourier-transform infrared (FTIR) study of AuNRs and AuNRs-LA-COS were summarized in Figure 1c. The AuNRs showed characteristic peaks at ~1490 cm^−1^ (CTAB). The FTIR spectra confirmed surface modification of AuNRs with COS, where the strong band at ~1490 cm^−1^ (CTAB) disappeared and peaks of the COS spectrum at 1649, 1570, 1162, and 1082 cm^−1^ were observed in relation to the C=O (carbonyl group) stretching of the secondary amide, NH_3_^+^ (amino group) bending vibrations, asymmetric C–O–C stretching, and C–O skeletal vibration.

**Figure 2:**
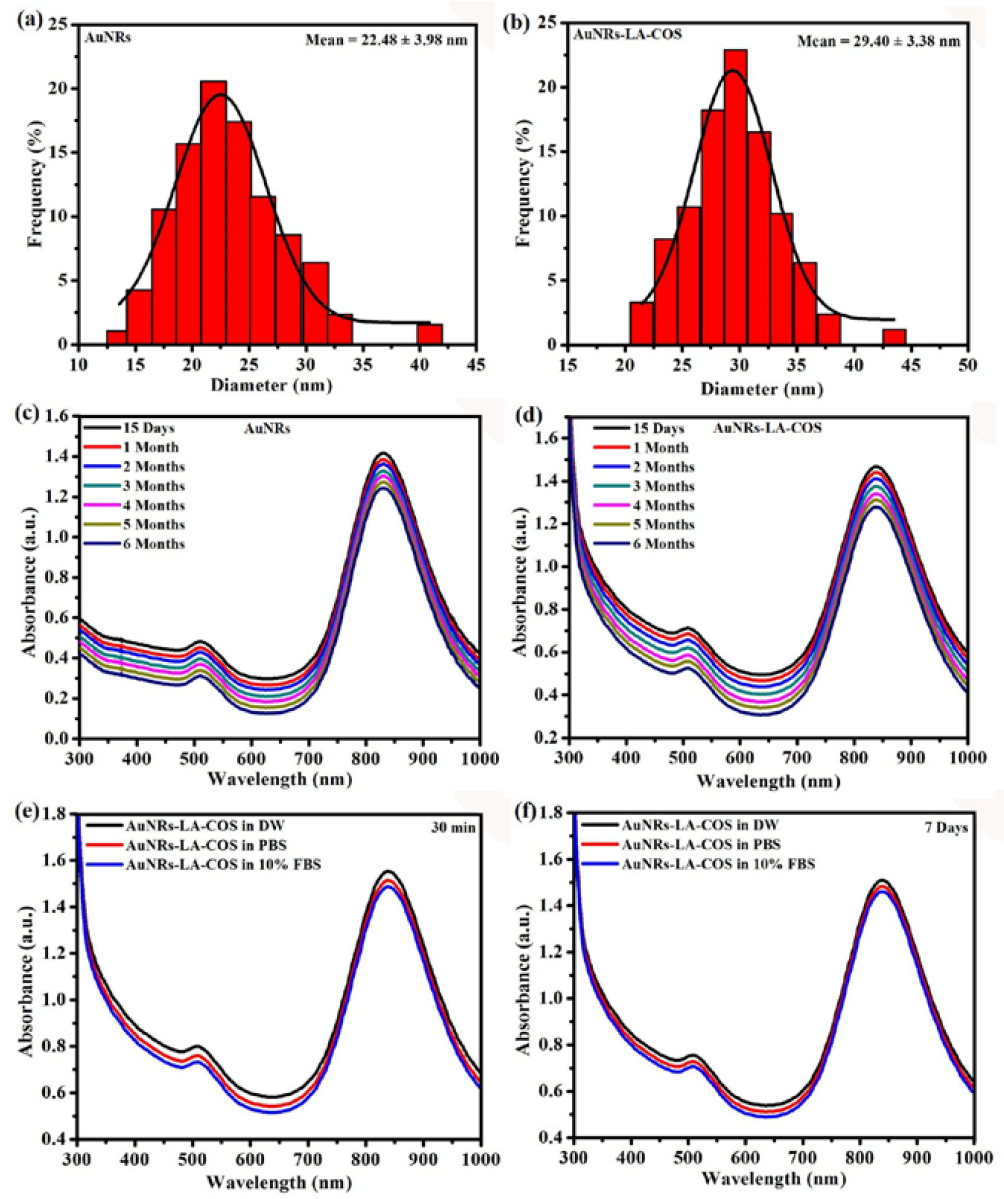
DLS results of AuNRs (**a**) and AuNRs-LA-COS (**b**). UV-Vis-NIR absorbance spectra of AuNRs (**c**) and AuNRs-LA-COS (**d**) of six months stability study. UV-Vis-NIR absorbance spectra of AuNRs-LA-COS of dispersion stability in DW, PBS, and DMEM supplemented with 10% fetal bovine serum (FBS) for 30 min (**e**) and 7 days (**f**).

The morphology and size of the AuNRs and AuNRs-LA-COS were characterized by field emission transmission electron microscopy (FETEM) (Figure 1d and Figure S2). FETEM images showed that AuNRs had a mean length of 24 ± 4.2 nm and a diameter of 5.4 ± 3.5 nm. The average length and diameter of the AuNRs-LA-COS were 26 ± 3.1 and 6.8 ± 1.7 nm, respectively. Figure S3 shows the selected area electron diffraction pattern (SAED) pattern of AuNRs and AuNRs-LA-COS, which clearly showed the characteristic ring in the crystalline diffraction pattern can be denoted to (111), (200), (220), and (311) crystalline facets, making concentric rings with sequences corresponding to a face-centered cubic (fcc) lattice gold phase. Furthermore, the elemental composition of AuNRs and AuNRs-LA-COS were analyzed by energy dispersive X-ray spectroscopy (EDX) (Figure S4). The EDX spectrum of AuNRs and AuNRs-LA-COS showed a significant signal of the gold (Au) element, confirming the formation of AuNRs. Presence of elemental Cu was caused by the copper grid used for FETEM studies. The hydrodynamic size obtained by dynamic light scattering (DLS) measurement of AuNRs-LA-COS (29.40 ± 3.38 nm) was increased compared to the AuNRs (22.48 ± 3.98 nm) since the COS layers covered on the AuNRs (Figure 2a,b). In addition, AuNRs had a positive zeta potential (ZP) of +34.47 mV due to the presence of a bilayer of CTAB on the nanorods surface. AuNRs-LA-COS had a positive ZP of +36.83 mV due to the surface modification of COS with amine groups.

### 3.3. Stability Studies

The stability of nanoparticles is one of the most important characteristics for therapeutic applications. The stability of AuNRs solutions was studied at different months with no observable changes in the UV-Vis spectra of the AuNRs (Figure 2c). The stability of AuNRs-LA-COS solutions were studied in different months, at various pH, and different concentration of NaCl. As shown in Figure 2d and Figure S5a,b, no noticeable changes in the UV-Vis spectra of the AuNRs-LA-COS were observed, which showed good stability of AuNRs-LA-COS at different months, under a variety of pH ranges, and NaCl. The stability of AuNRs-LA-COS solutions dispersed in DW, PBS, DMEM supplemented with 10% FBS were observed. The UV-Vis spectra of all the test solutions were taken after 30 min, 12 h, 24 h, 3 days, 5 days and 7 days of incubation at room temperature (Figure 2e,f and Figure S6a–d). The UV-Vis spectra of AuNRs-LA-COS in all solutions kept intended shape as prepared, with a typical short transverse plasmon band at 510 nm and a strong long longitudinal SPR band at 831 nm. The results suggested that the multidentate COS ligand could stabilize AuNRs with strong resistance to various conditions. In addition, the FETEM image of AuNRs-LA-COS showed its excellent shape and stability in PBS solution after 7 days (Figure S7). The hydrodynamic size of AuNRs-LA-COS in PBS solution before and after 7 days was 29.40 ± 4.51 and 28.76 ± 2.42 nm as measured by DLS (Figure S8).

### 3.4. Photothermal Conversion of AuNRs-LA-COS

**Figure 3:**
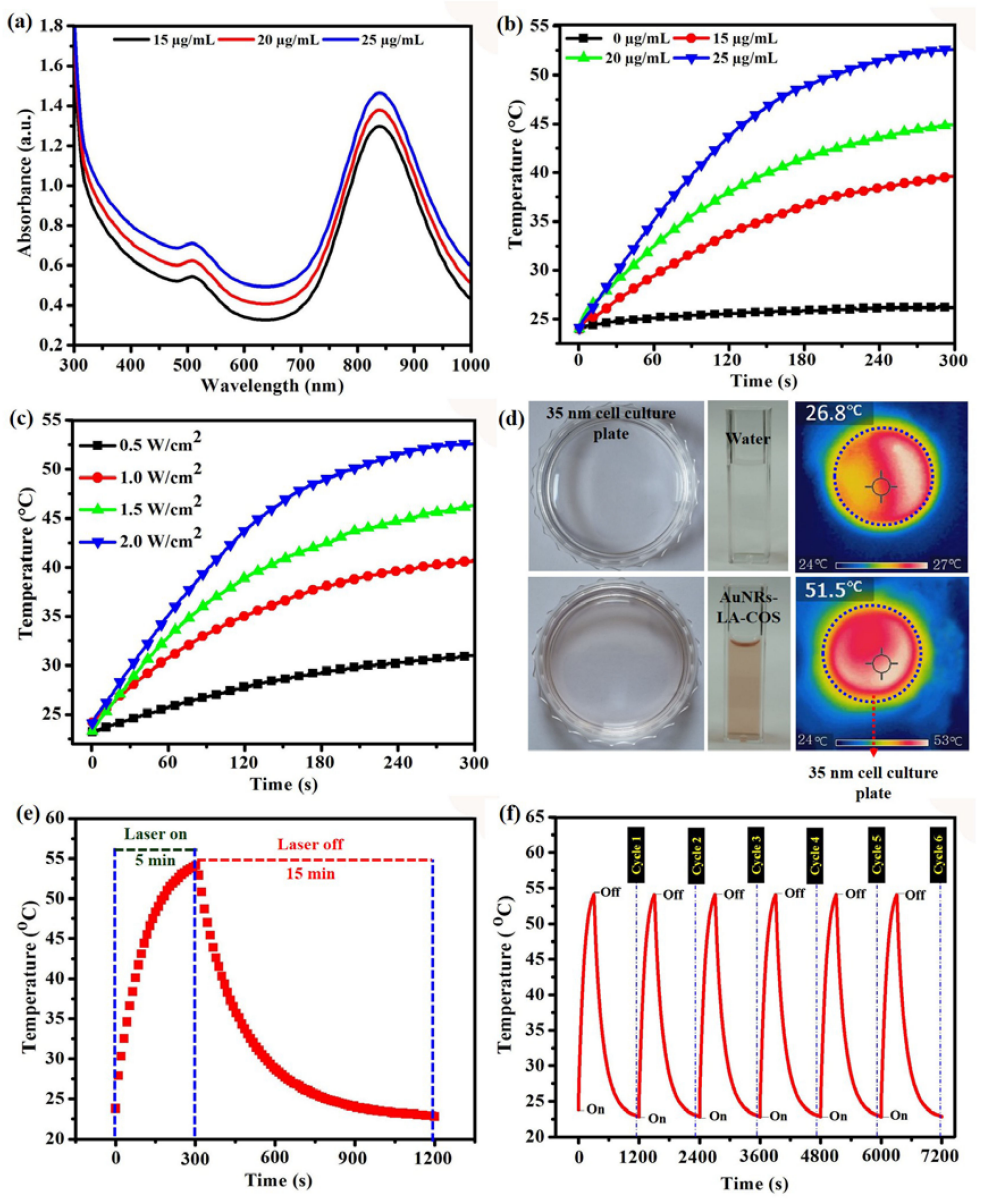
(**a**) UV-Vis-NIR absorbance spectra of different concentrations of AuNRs-LA-COS. (**b**) Temperature changes of different concentrations of AuNRs-LA-COS aqueous solutions under 808 nm NIR laser irradiation at 2.0 W/cm^2^ for 5 min. (**c**) Temperature changes of aqueous solution at a concentration of 25 µg/mL under 808 nm NIR laser irradiation at different power densities (0.5, 1.0, 1.5, and 2.0 W/cm^2^) for 5 min. (**d**) IR thermographs of water and AuNRs-LA-COS (25 µg/mL) solution in 35 mm cell culture plate under exposure to an 808 nm NIR laser irradiation at 2.0 W/cm^2^ for 5 min. (**e**) Heating and cooling curves of AuNRs-LA-COS (25 µg/mL). The laser lasted for 5 min, and then the laser was turned off for 15 min. (**f**) The change of temperature increase of AuNRs-LA-COS (25 µg/mL) for six laser on/off cycles NIR laser irradiation at 2.0 W/cm^2^.

Different concentrations of AuNRs-LA-COS (15, 20, and 25 µg/mL) were observed by UV-Vis spectroscopy (Figure 3a and Figure S9). The photothermal efficiency of different concentrations (15, 20, and 25 µg/mL) were assessed by the 808 nm NIR laser irradiation at different power densities (0.5, 1.0, 1.5, and 2.0 W/cm^2^) for 5 min (Figure 3b and Figure S10a–c). The results suggested that temperatures of all the AuNRs-LA-COS solutions increased with 808 nm NIR laser irradiation, and the aqueous solutions with higher concentrations and high power density exhibited a quick increase of temperature. In comparison, under the same conditions, the temperature of water showed no obvious increase. At the concentration of 25 µg/mL, the temperature rapidly reached 52.6 °C within 5 min of laser irradiation, suggesting that AuNRs-LA-COS can be easily heated up, above 50 °C, sufficient for photothermal treatment to kill tumor cells. The temperature of AuNRs-LA-COS (25 µg/mL) was evaluated by 808 nm NIR laser irradiation at different power densities (0.5, 1.0, 1.5, and 2.0 W/cm^2^) for 5 min and the aqueous solution had a temperature increase of 31.1, 40.7, 46.3, and 52.6 °C, respectively, indicating their great photothermal property (Figure 3c). Furthermore, temperature variations of all the solutions were recorded using a FLIR i5 infrared (IR) camera (Figure 3d and Figure S11a–d), which suggested that AuNRs-LA-COS were promising photothermal agents for cancer therapy.

To assess NIR photostability of AuNRs-LA-COS (25 µg/mL), six cycles of irradiation on/off with an 808 nm NIR laser irradiation were performed, and the aqueous solution of AuNRs-LA-COS irradiated with an 808 nm NIR laser at 2 W/cm^2^ for 5 min (laser on), then cooled to room temperature without irradiation for 15 min (laser off) (Figure 3e). After six cycles of the laser on/off, the temperature of AuNRs-LA-COS showed no obvious change (Figure 3f), also strong evidence proved high photostability of the AuNRs-LA-COS. The NIR photostability was further investigated using UV-Vis spectrum before and after irradiation. There were no noticeable changes in the UV-vis spectrum of the AuNRs-LA-COS (Figure S12), suggesting a highly stable nature. Furthermore, the FETEM image of AuNRs-LA-COS exhibited good shape and stability after six cycles of the laser on/off (Figure S13), suggesting colloidal stability of AuNRs-LA-COS. The hydrodynamic size of AuNRs-LA-COS before and after six cycles of the laser on/off was 29.40 ± 3.38 and 27.89 ± 4.51 nm as measured by DLS (Figure S14).

### 3.5. Biocompatibility Study

To explore the potential for biomedical applications, biocompatibility of AuNRs-LA-COS was first investigated. HEK 293 cells (a normal human embryonic kidney cell line) were incubated with different concentrations of AuNRs-LA-COS (10–100 µg/mL) for 24 h and 48 h and evaluated using an 3-(4,5-dimethylthiazol-2-yl)-2,5-diphenyltetrazolium bromide (MTT) assay to quantify cell viability (Figure S15). No cytotoxic effects of AuNRs-LA-COS were observed at the highest concentration of 100 µg/mL, confirming that AuNRs-LA-COS had excellent biocompatibility in the tested concentration range; the most important characteristic for biomedical applications.

### 3.6. In Vitro Cytotoxicity Study

To evaluate the biomedical application of AuNRs-LA-COS, we next tested their potential cytotoxicity. The MTT cell viability assay was used to assess the cytotoxicity of MDA-MB-231 cells (a human breast cancer cell line) incubated with different concentrations of AuNRs-LA-COS (10–100 µg/mL) for 24 h and 48 h (Figure S16). They showed significantly reduced proliferation in a dose- and time-dependent cytotoxicity, all concentrations of AuNRs-LA-COS showed no obvious cytotoxicity after 24 h and 48 h. These results suggested that AuNRs-LA-COS have less toxicity, suggesting their excellent suitability for cancer therapy.

### 3.7. In Vitro Photothermal Ablation of MDA-MB-231 Cells

**Figure 4:**
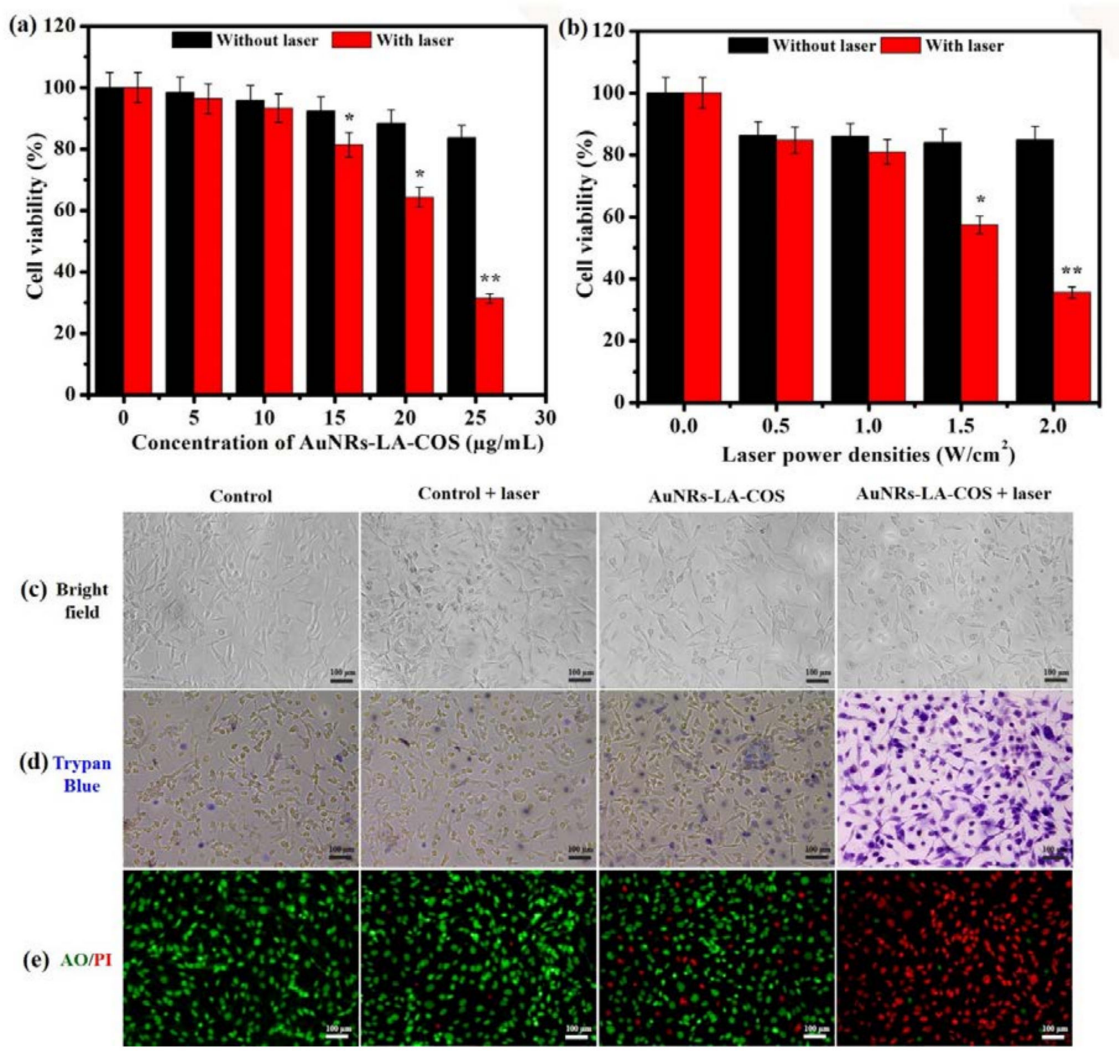
(**a**) Cell viability of MDA-MB-231 cells incubated with different concentration of AuNRs-LA-COS with or without 808 nm NIR laser irradiation at 2.0 W/cm^2^ for 5 min. (**b**) Cell viability of MDA-MB-231 cells treated with AuNRs-LA-COS (25 µg/mL) with 808 nm NIR laser irradiation at different power densities (0.5, 1.0, 1.5, and 2.0 W/cm^2^) for 5 min. (**c**) Morphological observation in MDA-MB-231 cells incubated with or without AuNRs-LA-COS with or without 808 nm NIR laser irradiation at 2.0 W/cm^2^ for 5 min (20× magnification). (**d**) Optical images stained by trypan blue and the optical image of MDA-MB-231 cells incubated with or without AuNRs-LA-COS (25 µg/mL), with or without 808 nm NIR laser irradiation at 2.0 W/cm^2^ for 5 min (20× magnification). (**e**) Fluorescence images stained by AO (live: green) and PI (dead: red). Merged fluorescence images of MDA-MB-231 cells treated with or without AuNRs-LA-COS (25 µg/mL) with or without 808 nm NIR laser irradiation at 2.0 W/cm^2^ for 5 min (20× magnification).

To assess the in vitro photothermal effect of AuNRs-LA-COS, an MTT assay was carried out using the MDA-MB-231 cells incubated with different concentrations of AuNRs-LA-COS (5, 10, 15, 20 and 25 µg/mL) under 808 nm NIR laser irradiation at 2 W/cm^2^ for 5 min. As shown in Figure 4a, the high concentration of AuNRs-LA-COS destroyed a large number of cells under 808 nm NIR laser irradiation at 2 W/cm^2^ for 5 min. Most of the cells were destroyed after incubation with 25 µg/mL of AuNRs-LA-COS and 808 nm NIR laser irradiation at 2 W/cm^2^ for 5 min compared with corresponding control experimental samples without laser irradiation. Furthermore, we next incubated MDA-MB-231 cells with 25 µg/mL of AuNRs-LA-COS for 4 h, then an 808 nm NIR laser irradiation for 5 min at different power densities (0.5, 1.0, 1.5, and 2.0 W/cm^2^) for cell ablation (Figure 4b). Most of the cancer cells were killed after incubation with AuNRs-LA-COS (25 µg/mL) and 808 nm NIR laser irradiation at 2 W/cm^2^ for 5 min, suggesting that AuNRs-LA-COS have great promise for photothermal ablation of cancer cells.

**Figure 5:**
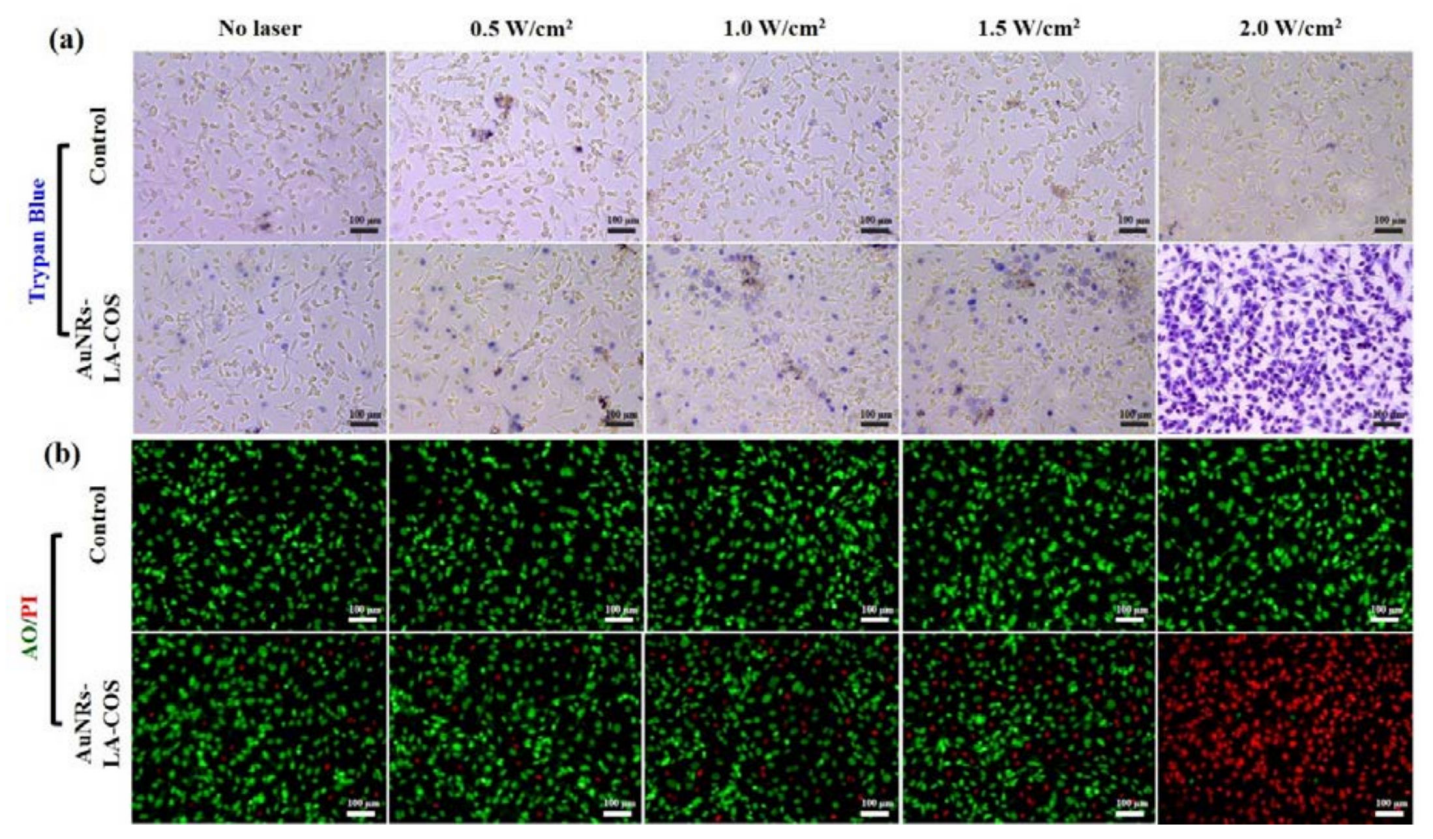
(**a**) Optical images stained by trypan blue and the optical image of MDA-MB-231 cells incubated with or without AuNRs-LA-COS (25 µg/mL) under 808 nm NIR laser irradiation at different power densities (0.5, 1.0, 1.5, and 2.0 W/cm^2^) for 5 min (20× magnification). (**b**) Merged fluorescence images of MDA-MB-231 cells treated with or without AuNRs-LA-COS (25 µg/mL), with or without 808 nm NIR laser irradiation at different power densities (0.5, 1.0, 1.5, and 2.0 W/cm^2^) for 5 min (20× magnification).

The photothermal efficiency of AuNRs-LA-COS was further assessed by light microscopic observation of cell morphology. The MDA-MB-231 cells treated with laser irradiation alone or with 25 µg/mL of AuNRs-LA-COS without 808 nm NIR laser irradiation had similar cell morphology and cellular shape almost similar to the PBS control. In contrast, when the MDA-MB-231 cells were treated with 25 µg/mL of AuNRs-LA-COS and 808 nm NIR laser irradiation at 2 W/cm^2^ for 5 min, most of the cells were killed, indicating that AuNRs-LA-COS were able to successfully ablate cancer cells under NIR laser irradiation (Figure 4c). We next assessed the photothermal effect of AuNRs-LA-COS to ablate cancer cells by trypan blue staining under 808 nm NIR laser irradiation. The control groups of cells were treated with or without 808 nm NIR laser irradiation, no noticeable changes in the control groups were observed (Figure 4d). When the cells were treated with 25 µg/mL of AuNRs-LA-COS without 808 nm NIR laser irradiation, only a few dead cells were observed. In contrast, cells incubated with 25 µg/mL of AuNRs-LA-COS after laser irradiation were stained blue, owing to the ablation of cancer cells by 808 nm NIR laser irradiation. Furthermore, the cells incubated with 25 µg/mL of AuNRs-LA-COS for 4 h, then irradiated with NIR laser at 808 nm for 5 min, at different power densities (0.5, 1.0, 1.5, and 2.0 W/cm^2^) were used for cell ablation studies. As shown in Figure 5a, a majority of cells were killed after incubation with AuNRs-LA-COS (25 µg/mL) and 808 nm NIR laser irradiation at 2 W/cm^2^ for 5 min, indicating the combination was cytotoxic to cancer cells.

**Figure 6:**
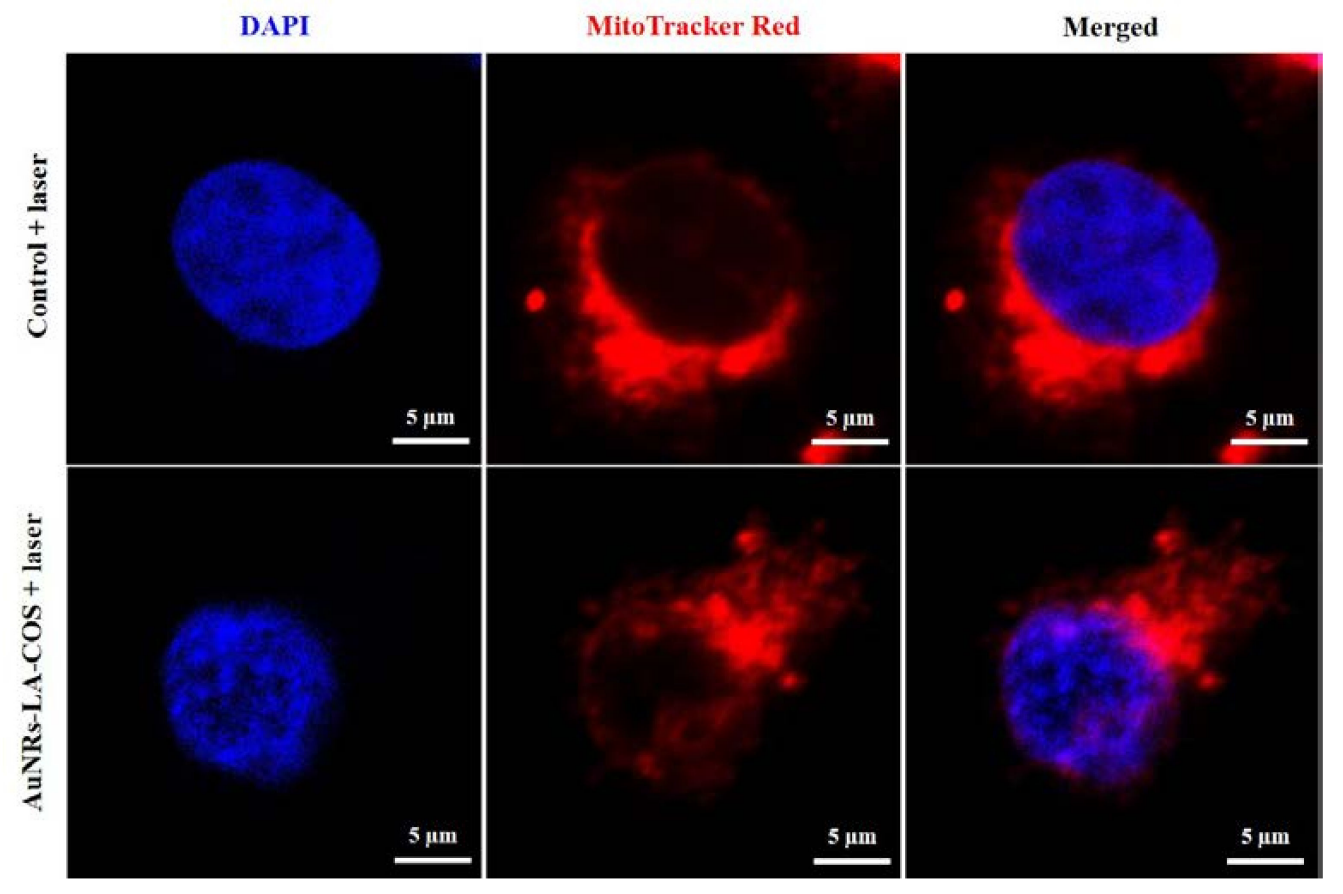
Confocal fluorescence microscope images stained by DAPI and MitoTracker Red. Confocal fluorescence microscope image of MDA-MB-231 cells treated without or with 25 µg/mL AuNRs-LA-COS and irradiated with 808 nm NIR laser at 2 W/cm^2^ for 5 min.

The photothermal effect of cancer cells was further confirmed by fluorescent microscopy images of live–dead cell staining. After the NIR laser irradiation, AO and PI were used to stain the cancer cells to differentiate live (green fluorescence) and dead (red fluorescence) cells. The results suggested that most of the MDA-MB-231 cells were killed after incubation with AuNRs-LA-COS (25 µg/mL) after 808 nm NIR laser irradiation at 2 W/cm^2^ for 5 min, whereas very few cells were destroyed in the three control groups (Figure 4e). In addition, the cells were treated with AuNRs-LA-COS (25 µg/mL) with or without 808 nm NIR laser irradiation for 5 min at different power densities (0.5, 1.0, 1.5, and 2.0 W/cm^2^). When the laser power density increased to 2.0 W/cm^2^, complete cell death was observed (Figure 5b). The ability of AuNRs-LA-COS for photothermal ablation in cancer cells was further confirmed using confocal laser scanning microscopy. The cells were treated with or without AuNRs-LA-COS (25 µg/mL) and irradiated with 808 nm NIR laser at 2 W/cm^2^ for 5 min. After the laser irradiation, DAPI and MitoTracker Red or DAPI and LysoTracker green were used to stain the cancer cells. DAPI was used to specifically stain the nucleus. MitoTracker Red and LysoTracker green were also used for staining mitochondria and lysosomes, respectively. DAPI clearly observed fragmented nuclei in the AuNRs-LA-COS (25 µg/mL) COS under 808 nm NIR laser irradiation at 2.0 W/cm^2^ for 5 min whereas the control cells showed normal nuclei. The cells incubated with or without 25 µg/mL of AuNRs-LA-COS after 808 nm NIR laser irradiation were co-localized with mitochondria and lysosomes labeled with fluorescence dye MitoTracker Red and LysoTracker green in the cytoplasm compared to the control cells (Figure 6 and Figures S17–S19).

To explain cell death mode after photothermal effect, the MDA-MB-231 cells were further evaluated by apoptosis assay using flow cytometry. As shown in Figure S20a, the cells were treated with or without 25 µg/mL of AuNRs-LA-COS for 4 h, with or without 808 nm NIR laser irradiation at 2 W/cm^2^ for 5 min, and then double-labeled with Annexin V-FITC and PI successively. These results showed that AuNRs-LA-COS and laser irradiation induced significant apoptosis (63.3%) compared with the three control groups (0.38%, 1.74%, and 12.01%) (Figure S20b), indicating that the cytotoxic effect of AuNRs-LA-COS on cancer cells could clearly be promoted by laser irradiation.

### 3.8. In Vivo Photothermal Ablation of MDA-MB-231 Cells

**Figure 7:**
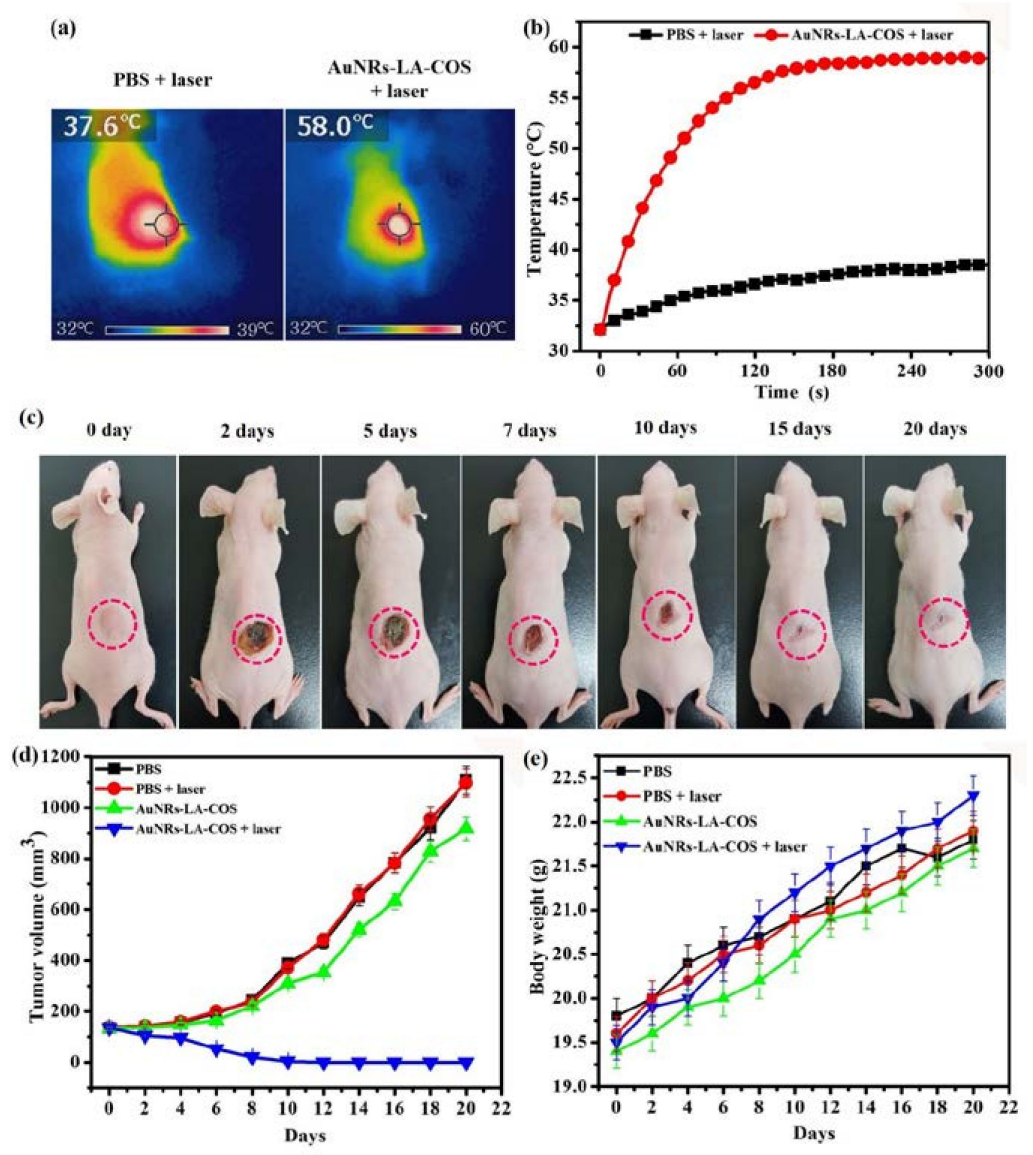
(**a**) IR thermographs of mice injected with the PBS and AuNRs-LA-COS and irradiated with an 808 nm NIR laser at 2 W/cm^2^ for 5 min. Thermographs were recorded with an infrared thermal camera as function of irradiation time. (**b**) Temperature changes of tumor-bearing mice after intratumoral injection with PBS and AuNRs-LA-COS with an 808 nm NIR laser at 2 W/cm^2^ for 5 min. (**c**) Photographs of mice taken before treatments (0 day) and 2, 5, 7, 10, 15, and 20 days after treatments of AuNRs-LA-COS with an 808 nm NIR laser at 2 W/cm^2^ for 5 min. (**d**) The relative tumor volume in different mice groups after photothermal therapy. Data is expressed as mean ± SD of the three experiments. (**e**) Body weight of tumor-bearing mice among different treatment groups after NIR laser irradiation. Data is expressed as mean ± SD of the three experiments.

The in vivo photothermal ablation of AuNRs-LA-COS was further evaluated in tumor-bearing mice. When their tumor sizes reached approximately 130 mm^3^ the mice were injected with an intratumoral injection of either sterilized PBS solution or AuNRs-LA-COS. The animals were randomly divided into four groups and treated with PBS only, PBS + 808 nm NIR laser irradiation, AuNRs-LA-COS only, and AuNRs-LA-COS + 808 nm NIR laser irradiation, respectively. We used an IR thermal camera to record the local temperature difference (Figure 7a). As shown in Figure 7b, the temperature of tumor center in mice treated with AuNRs-LA-COS increased from 32.1 to 58.9 °C during 808 nm NIR laser irradiation at 2 W/cm^2^ for 5 min, which is sufficiently high to ablate the tumor cells. However, the temperature of tumor center in mice treated with PBS only displayed a minimal increase to 38.5 °C at the same conditions, insufficient to kill the tumor.

We measured the tumor size every day after treatment (Figure 7d). The results suggested that tumors treated with AuNRs-LA-COS under 808 nm laser irradiation at 2 W/cm^2^ for 5 min were able to be completely destroyed. On days 20, the tumor tissue almost completely disappeared, the scars finally disappeared and were reconstructed with normal tissues (Figure 7c and Figure S21), suggesting that the combination of AuNRs-LA-COS and laser irradiation was important for efficient PTT of tumors. In addition, the four groups of treated mice maintained their body weights during the experimental period, indicating that PBS only, PBS + laser irradiation, AuNRs-LA-COS only, or the combination of the AuNRs-LA-COS + laser irradiation were unable to generate toxicity to the mice (Figure 7e). At the 20th day of treatment, animals were sacrificed and tumors removed (Figure S22a). The tumor weights of group IV were the lowest compared with the three control groups (Figure S22b), suggesting that AuNRs-LA-COS under laser irradiation could completely ablate tumors in mice.

The biodistribution of AuNRs-LA-COS in major organs (heart, kidney, spleen, lung, and liver) and the tumor, were evaluated using tumor-bearing mice. Major organs and tumor tissues were harvested at 24 h and 20 days post-injection of AuNRs-LA-COS and the gold (Au) content was measured by ICP-MS (Figure S23). The majority of AuNRs-LA-COS accumulated in the tumor at 24 h and the content of Au element attained 19.38% ID/g (injected dose/g). The tumor tissue completely disappeared at 20 days post-injection of AuNRs-LA-COS. Furthermore, the Au was mainly found in liver and spleen at 24 h and 20 days and then cleared from the body after 5 days, which guaranteed the safety of the AuNRs-LA-COS in the body.

**Figure 8:**
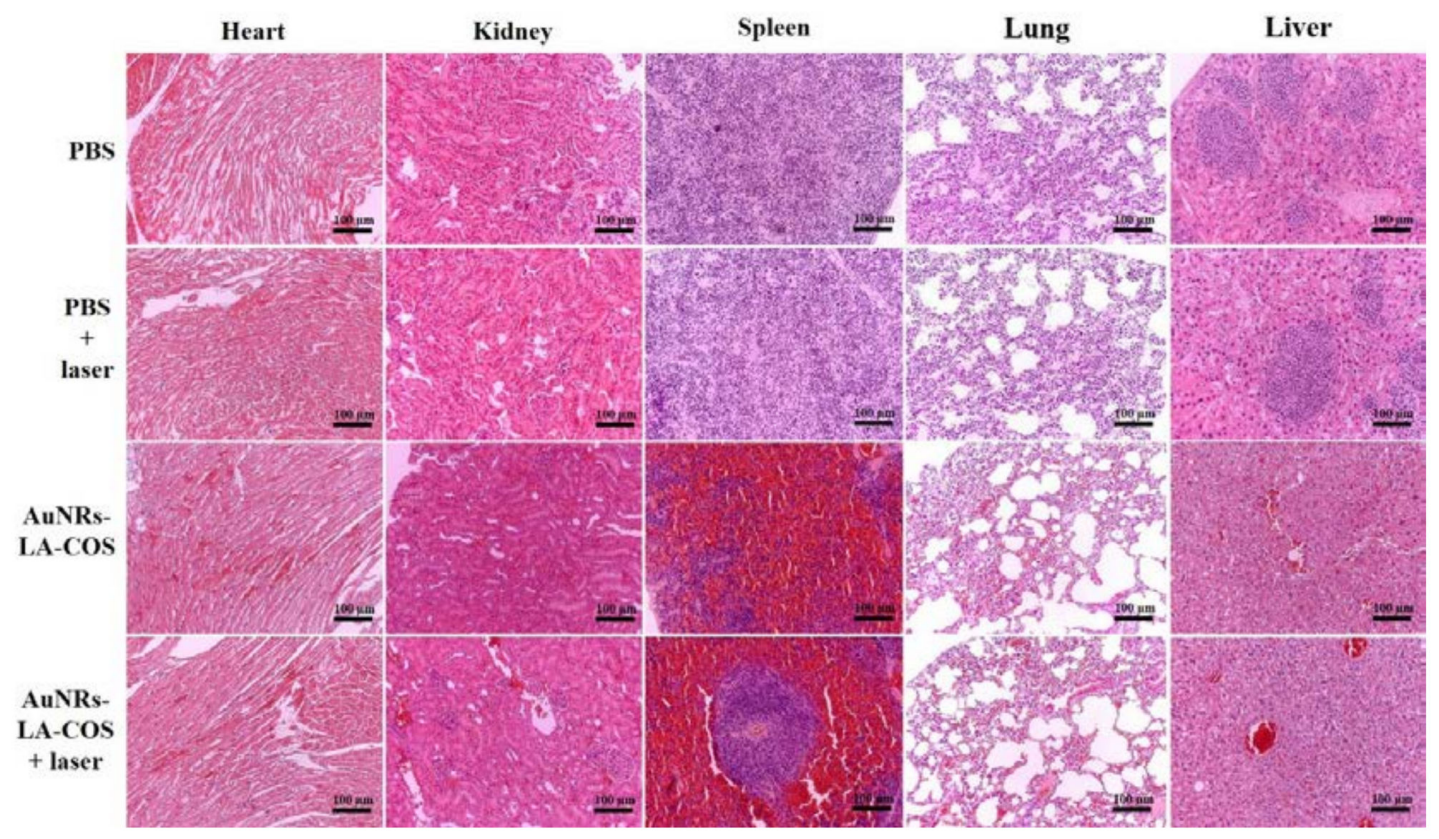
Histology staining of major organs collected from different groups of mice 20 days after NIR laser irradiation.

The photothermal ablation of tumors using AuNRs-LA-COS was further confirmed by H&E staining (Figure S24). The tumor tissues were collected, fixed, and sectioned at 1 and 4 days after laser irradiation. As expected, the H&E staining exhibited that a majority of the tumor cells lost their membrane integrity, resulting in notable cellular necrosis in tumor sections treated with AuNRs-LA-COS + NIR laser irradiation. H&E staining of the tumor sections exhibited that tumors treated with PBS + NIR laser irradiation showed well-shaped tumor cells, without the appearance of necrosis regions. After 4 days, tumor tissues slowly disappeared and no tumor regrowth was observed in the AuNRs-LA-COS + laser irradiation. At days 20 of treatment, the tumor tissue fully inhibited and normal tissues reconstructed. At day 20 of treatment, the mice were sacrificed and major organs (heart, kidney, spleen, lung, and liver) harvested for H&E staining. As shown in Figure 8, no apparent histopathological abnormalities or lesions were observed in the AuNRs-LA-COS treated mice. Therefore, these results suggested that no significant organ damage in the AuNRs-LA-COS treated groups were detected, indicating that AuNRs-LA-COS have no noticeable long-term toxicity to mice in vivo.

## 4. Conclusions

In summary, we have successfully designed and fabricated novel agents for photothermal ablation of cancer cells. AuNRs-LA-COS have attracted considerable research attention for therapeutic applications because of their excellent biocompatibility, photothermal stability, and strongly enhanced absorption and scattering in NIR regions. AuNRs-LA-COS have high photothermal conversion, amenable for PTT of cancer cells in vitro and in vivo. Most importantly, after intratumoral injection of AuNRs-LA-COS into tumor-bearing mice, followed by 808 nm NIR laser irradiation, the tumor completely disappeared and normal tissues finally reconstructed, as a result of the excellent photothermal effects, with no regrowth observed over the therapeutic period of 20 days. To the best our knowledge, this is the first report on the potential use of AuNRs-LA-COS as novel agents for photothermal therapy. The combination of AuNRs-LA-COS and NIR laser irradiation in our strategy paves a new way for breast cancer therapy and holds great promise in the future.

## Supplementary Materials

The following are available online at http://www.mdpi.com/2073-4360/10/3/232/s1.
