# Supplementary material for: Biocompatible Chitosan Oligosaccharide Modified Gold Nanorods as Highly Effective Photothermal Agents for Ablation of Breast Cancer Cells
Source: Polymers (Basel). 2018 Feb 26;10(3):232. doi: 10.3390/polym10030232 (PMC6415155; doi:10.3390/polym10030232)
Supplement: Supplementary file 1 [file polymers-10-00232-s001.pdf]

Supplementary Materials: Biocompatible chitosan oligosaccharide modified gold nanorods as highly effective photothermal agents for ablation of breast cancer cells

Panchanathan Manivasagan [1], Subramaniyan Bharathiraja [1], Madhappan Santha Moorthy [1], Sudip Mondal [1], Thanh Phuoc Nguyen [2], Hyehyun Kim [1], Thi Tuong Vy Phan [1], Kang Dae Lee [3], Junghwan Oh [1,2] *

[1] Marine-Integrated Bionics Research Center, Pukyong National University, Busan 48513, Republic of Korea; manimaribtech@gmail.com (P. M); sbrbtc@gmail.com (S. B); santham83@gmail.com (M. S. M); mailsudipmondal@gmail.com (S. M); hyki7732@colorado.edu (H. K); phanvy120690@gmail.com (T. T. V. P); jungoh@pknu.ac.kr (J. O)
[2] Department of Biomedical Engineering and Center for Marine-Integrated Biotechnology (BK21 Plus), Pukyong National University, Busan 48513, Republic of Korea; ntphuoc2000@gmail.com (T. P. N); jungoh@pknu.ac.kr (J. O)
[3] Department of Otolaryngology Head and Neck Surgery, Kosin University Gospel Hospital, Kosin University College of Medicine, 262 Gamcheon-ro, Seo-Gu, Busan 602-702, Republic of Korea; kdlee59@gmail.com
* Correspondence: jungoh@pknu.ac.kr (J. O); Tel.: +82-51-629—5771.

1. Experimental section

1.1. Materials

Chitosan oligosaccharide (COS), lipoic acid (LA), 1-(3-dimethylaminopropyl)-3-ethylcarbodiimide hydrochloride (EDC·HCl), *N*-hydroxysulfosuccinimide sodium (sulfo-NHS), gold (III) chloride trihydrate ($HAuCl_4 \cdot 3H_2O$), cetyltrimethylammonium bromide (CTAB), L-ascorbic acid, sodium borohydride ($NaBH_4$), silver nitrate ($AgNO_3$), 3-(4,5-dimethylthiazol-2-yl)-2,5-diphenyltetrazolium bromide (MTT), acridine orange (AO), propidium iodide (PI), DAPI (4',6-diamidine-2'-phenylindole dihydrochloride), and other biological reagents were purchased from Sigma–Aldrich Co. (St. Louis, MO, USA). 2,4,6-trinitrobenzene sulfonic acid (TNBS) was purchased from Thermo Fisher Scientific (Rockford, IL, USA). MitoTracker Red

was obtained from Invitrogen (Carlsbad, CA, USA). Fluorescein isothiocyanate Annexin V Apoptosis Detection Kit was obtained from BD Biosciences (USA).

1.2. *Characterization*

The absorption spectra of AuNRs solution were recorded on a Beckman DU 640 spectrophotometer (Beckman coulter, Fullerton, CA, USA) using quartz cuvettes with a 1 cm path length. The samples were determined by powder X-ray diffraction (XRD) with an X'Pert-MPD PW 3050 diffractometer (Phillips, The Netherlands). Fourier-transform infrared (FTIR) spectra were obtained on a Spectrum 100 FTIR spectrometer (PerkinElmer, USA). The morphologies of the samples were measured by field emission transmission electron microscopy (FETEM) and selected area electron diffraction pattern (SAED) conducted with a JEM-2100F field emission transmission electron microscope (JEOL Ltd., Tokyo, Japan) at an accelerating voltage of 200 kV. The elemental components were examined by an energy dispersive X-ray spectroscopy (EDX) analyzer as the FETEM accessory. The dynamic light scattering (DLS) and zeta potential (ZP) results were measured using an electrophoretic light scattering spectrophotometer (ELS-8000, OTSUKA Electronics Co. Ltd., Japan).

1.3. *Cell culture*

A human embryonic kidney cell line (HEK 293) and human breast cancer cell line (MDA-MB-231) were provided by the Korean Cell Line Bank. The cells were cultivated in Dulbecco's modified Eagle's medium (DMEM; HyClone, Logan, Utah, USA) supplemented with 10% FBS (HyClone) and 1% penicillin-streptomycin (Corning, USA) under conditions of 95% humidity at 37 °C and 5% CO_2 atmosphere.

1.4. *Biocompatibility study*

HEK 293 cells were seeded into 96-well plates at a density of 1×10^4 cells/well and permitted to adhere overnight. The cells were treated with different concentrations of AuNRs-LA-COS (10 to 100 μg/mL) and

the plate was further incubated at 37 °C for 24 and 48 h. The cells were incubated with 0.5 mg/mL MTT in DMEM for 4 h in dark and then dissolved in dimethyl sulfoxide (DMSO) after the supernatant was discarded. Absorbance was measured on a microplate reader (BioTek, PowerWave XS2, Vermont, USA) at 540 nm.

1.5. Biodistribution studies

The mice were sacrificed at 24 h and at 20 days post-injection of AuNRs-LA-COS. Their heart, kidney, spleen, lung, liver, and tumor tissues were harvested and fully digested with 8 mL of aqua regia for gold (Au) content determination, using inductively coupled plasma mass spectrometry (ICP-MS, Nexion 300D, PerkinElmer, USA).

1.6. Statistical analysis

Data were expressed as the mean ± standard deviation from three independent experiments. The statistically significant difference between groups were determined by one-way analysis of variance in the SPSS software version 14.0 (SPSS Inc., Chicago, IL, USA).

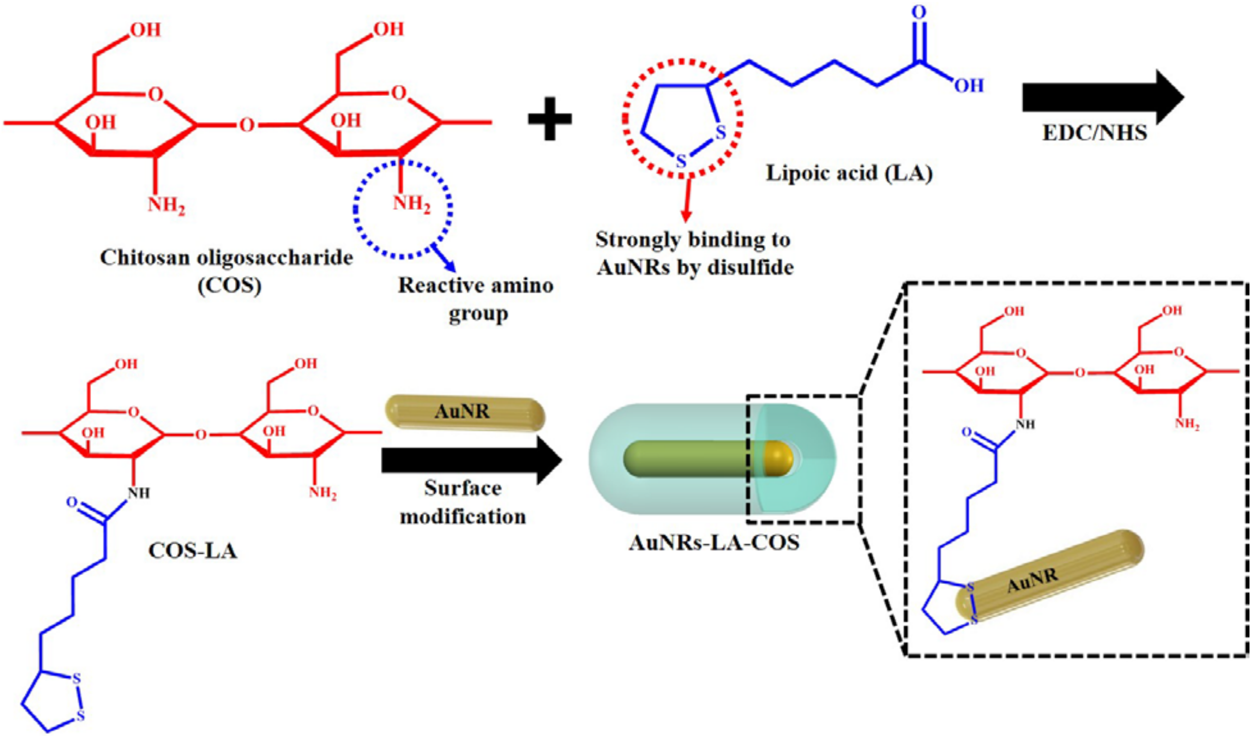

Figure S1. A schematic procedure for the preparation of AuNRs-LA-COS

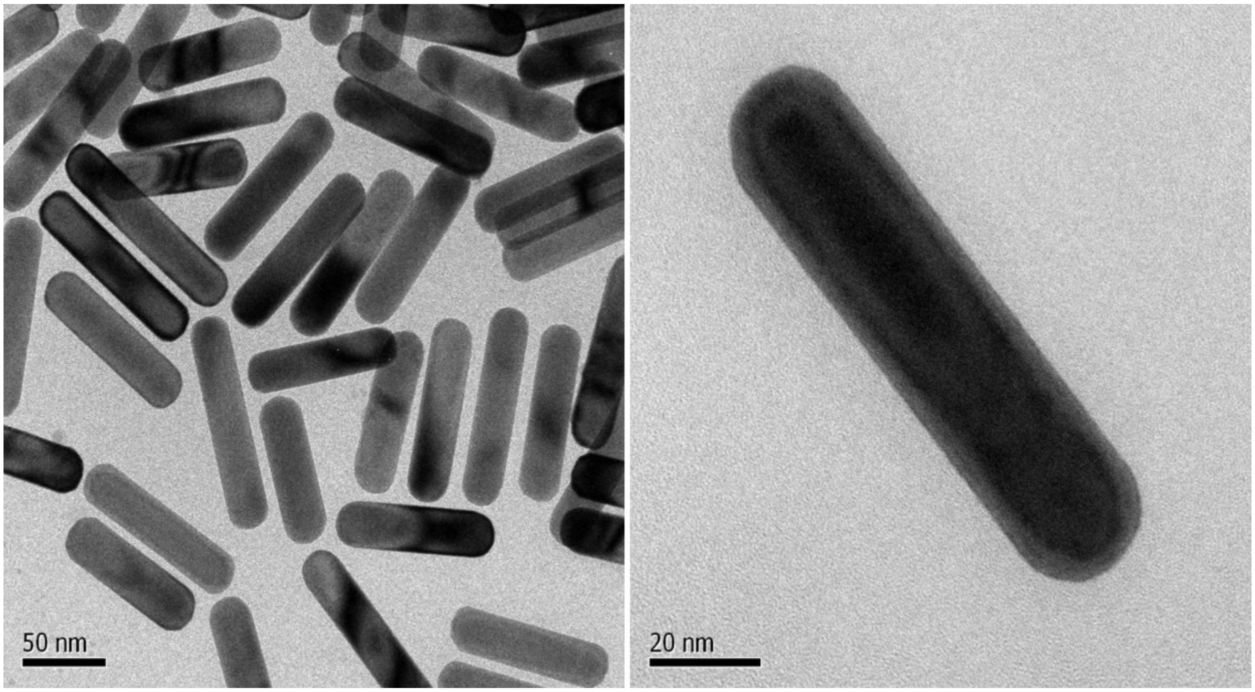

Figure S2. FETEM image of AuNRs

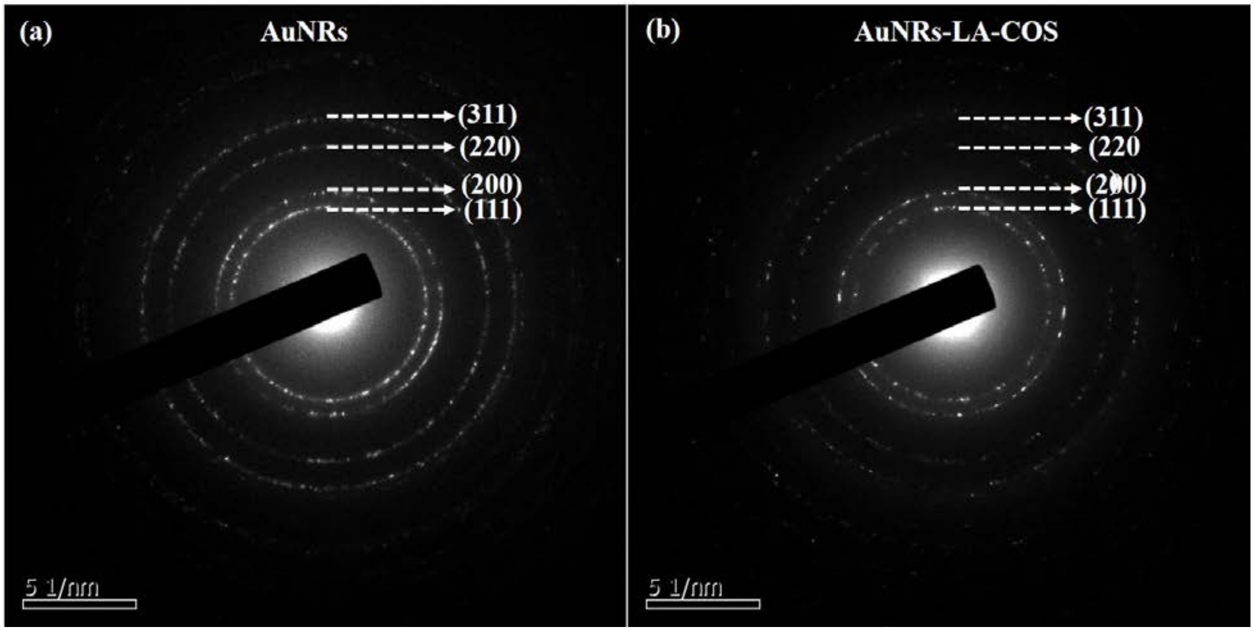

Figure S3. Selected area electron diffraction pattern (SAED) of AuNRs (a) and AuNRs-LA-COS (b).

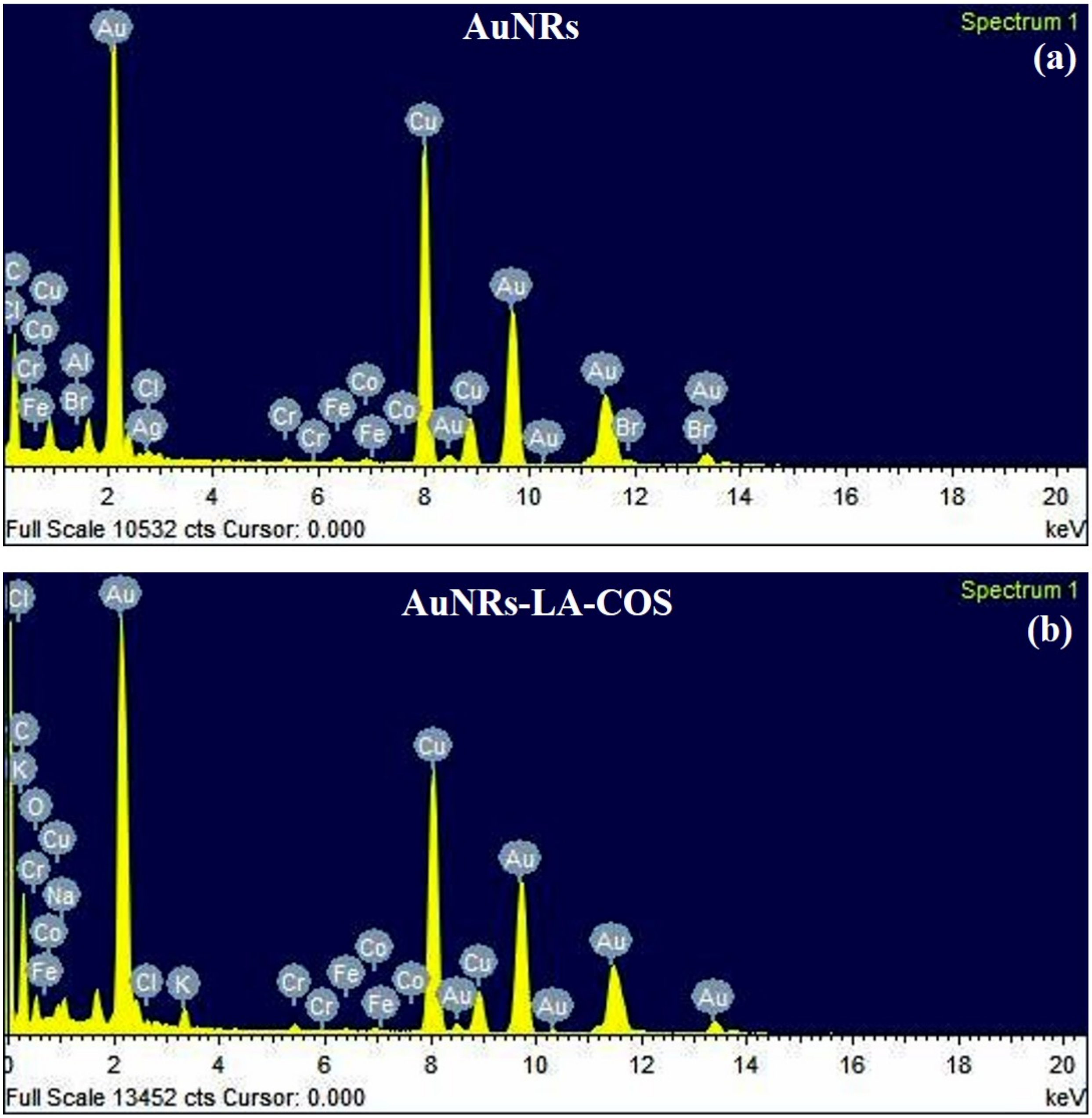

Figure S4. Energy-dispersive X-ray spectrum of AuNRs (a) and AuNRs-LA-COS (b).

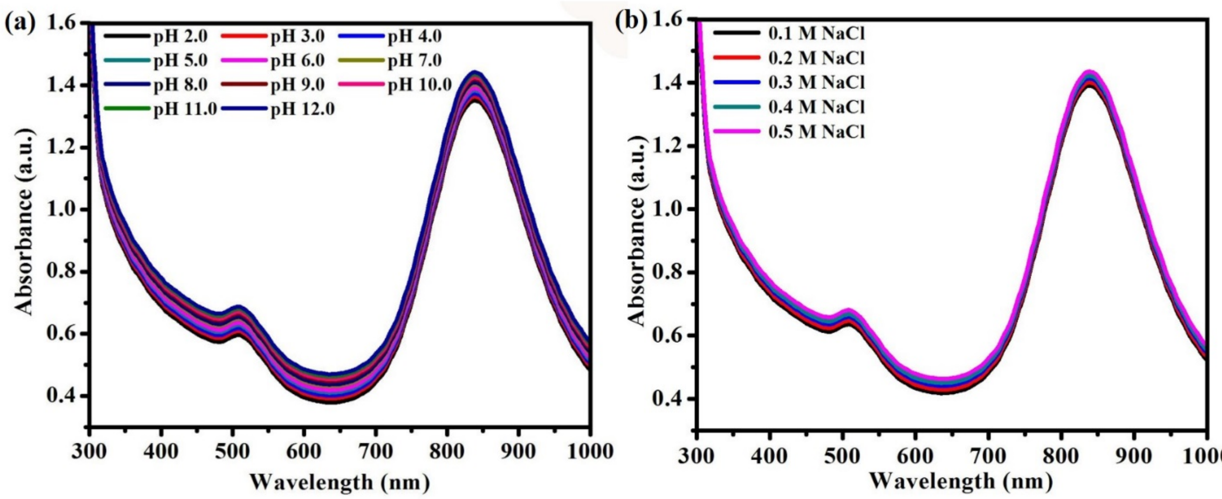

Figure S5. UV-Vis-NIR absorbance spectra of AuNRs-LA-COS at various pH (a) and different concentration of NaCl (b).

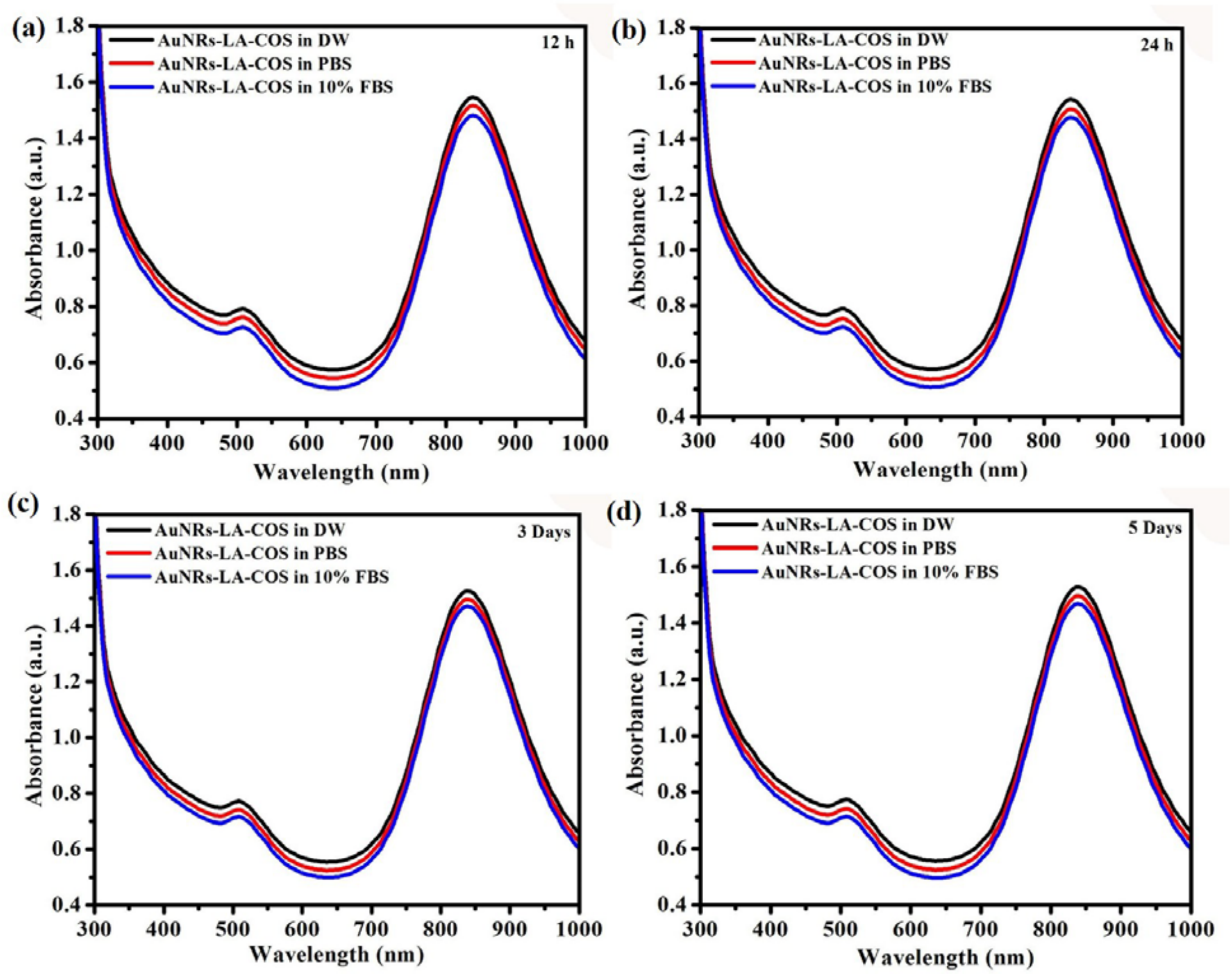

Figure S6. UV-Vis-NIR absorbance spectra of AuNRs-LA-COS of dispersion stability in distilled water (DW), PBS, DMEM supplemented with 10% fetal bovine serum (FBS) for 12 h (a), 24 h (b), 3 days, (c) and 5 days (d).

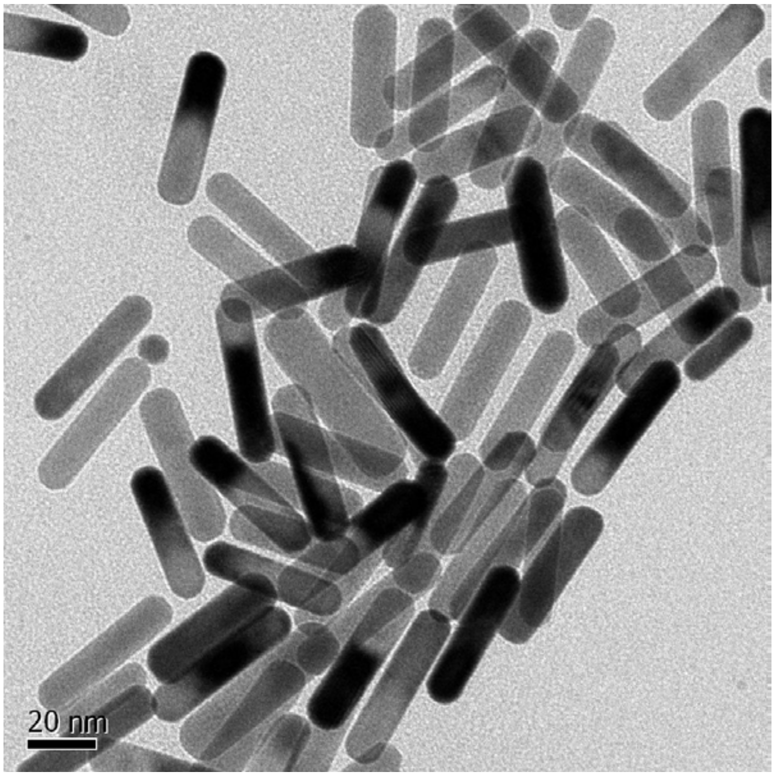

Figure S7. FETEM image of AuNRs-LA-COS of stable in PBS after 7 days.

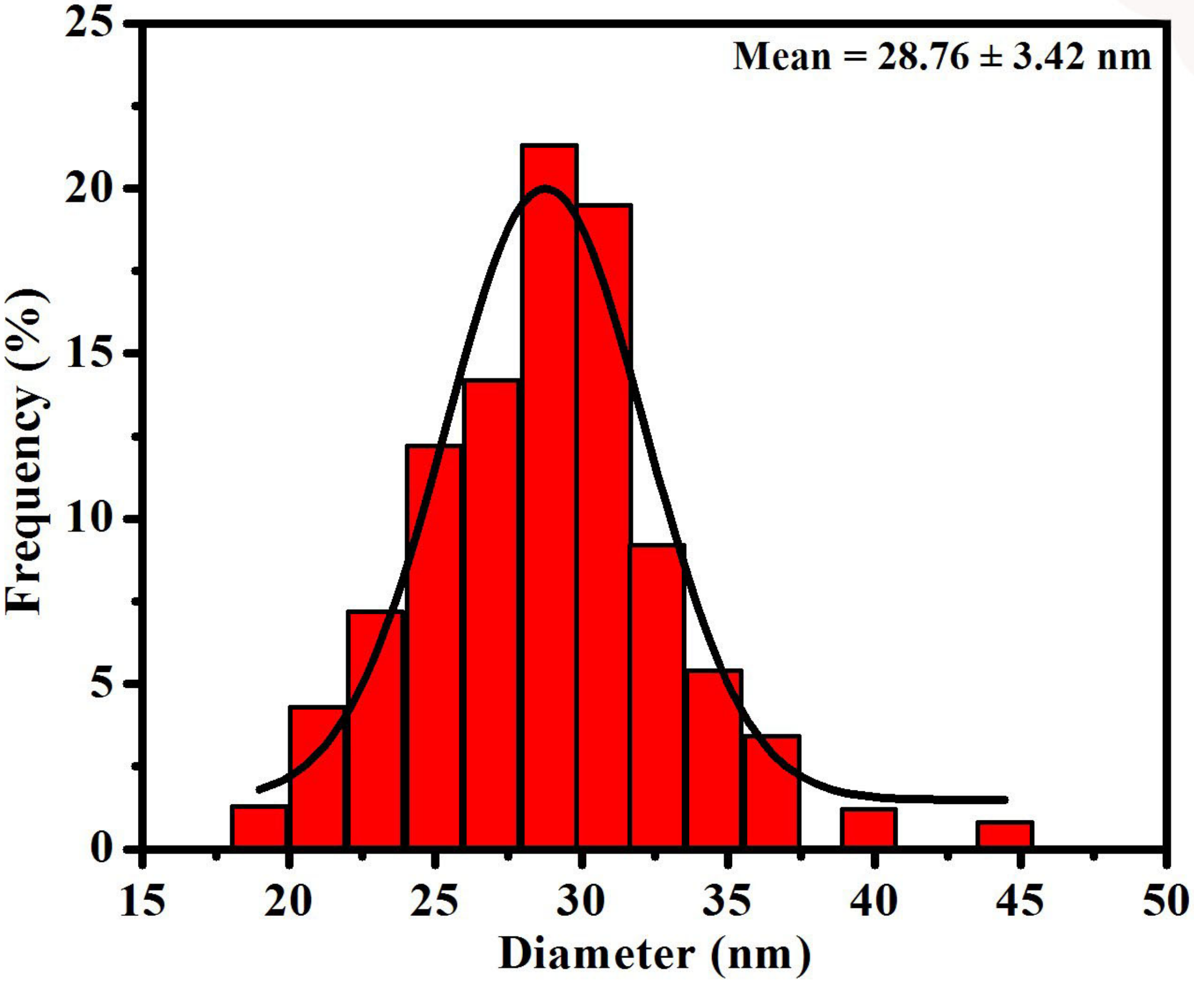

Figure S8. DLS results of AuNRs-LA-COS of stable in PBS solution after 7 days.

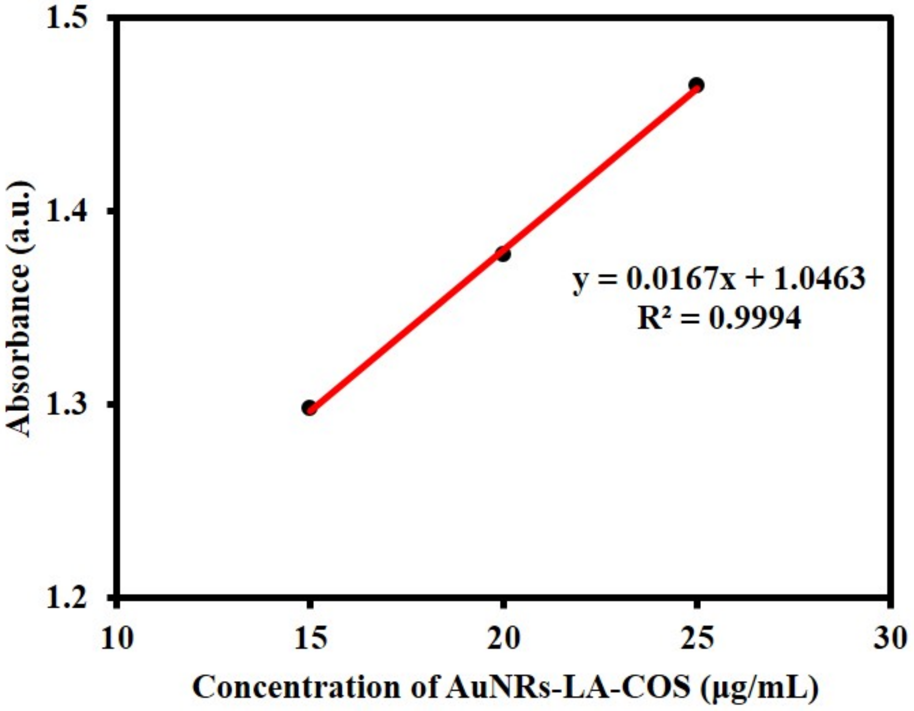

Figure S9. A linear relationship for the absorbance at 808 nm wavelength as a function of the concentration.

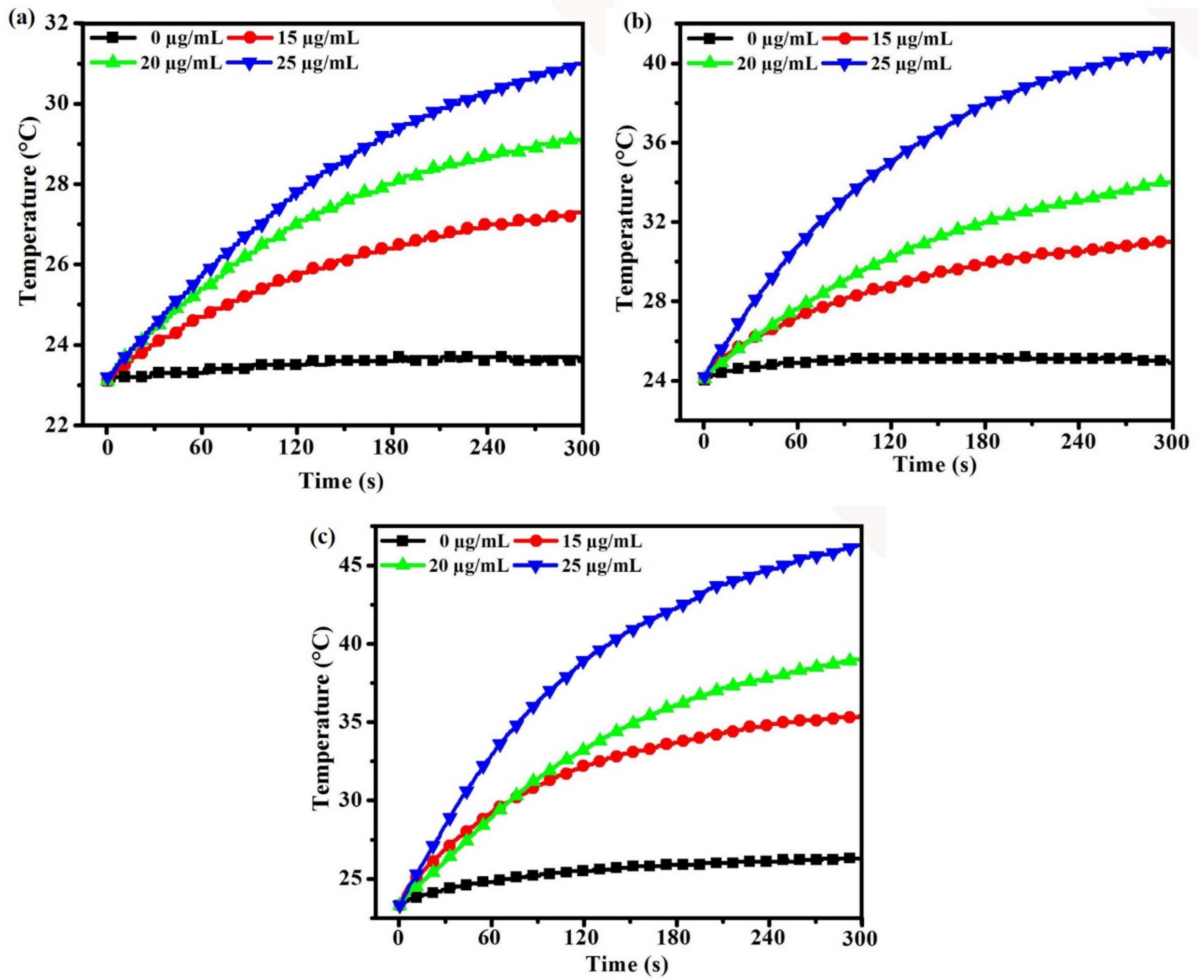

Figure S10. Temperature changes of different concentrations of AuNRs-LA-COS aqueous solutions under 808 nm NIR laser irradiation at different power densities (0.5 (a), 1.0 (b), and 1.5 W/cm² (c)) for 5 min.

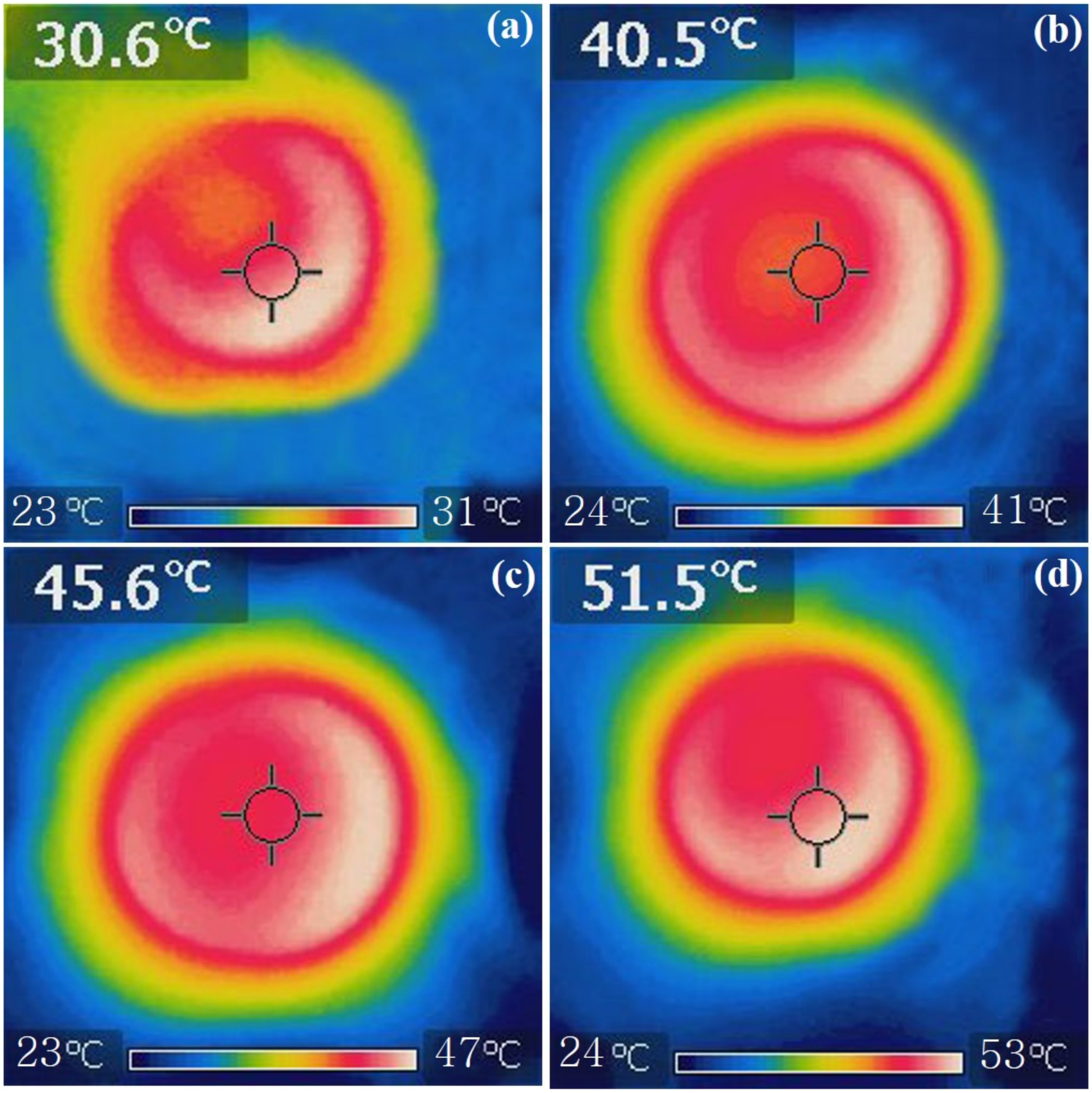

Figure S11. IR thermographs of AuNRs-LA-COS (25 μg/mL) solution in 35 mm cell culture plate under exposure to an 808 nm NIR laser irradiation at different power densities (0.5 (a), 1.0 (b), 1.5 (c), and 2.0 W/cm^2 (d)) for 5 min.

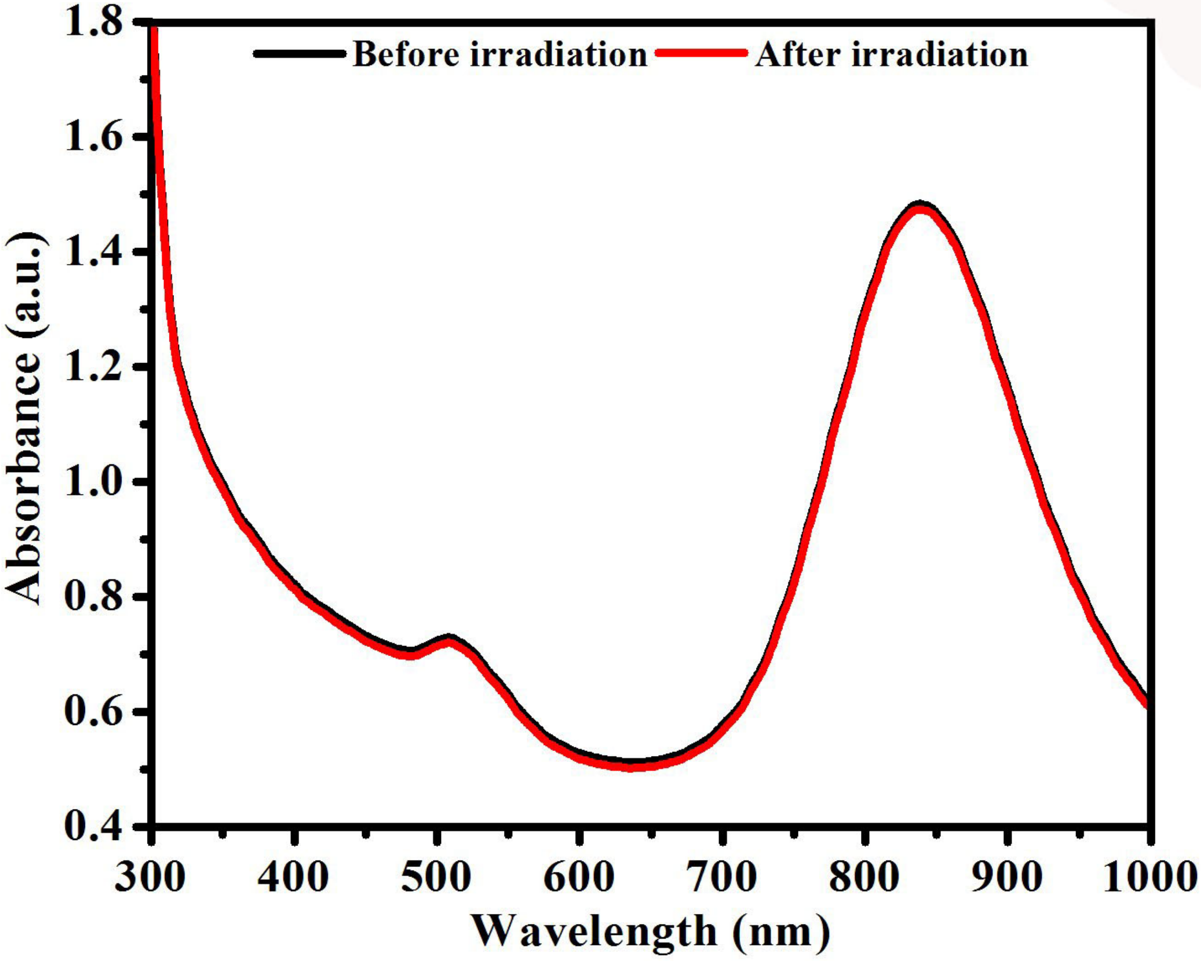

Figure S12. UV-Vis-NIR absorbance spectrum of AuNRs-LA-COS solution before and after six cycles of laser on/off NIR laser irradiation.

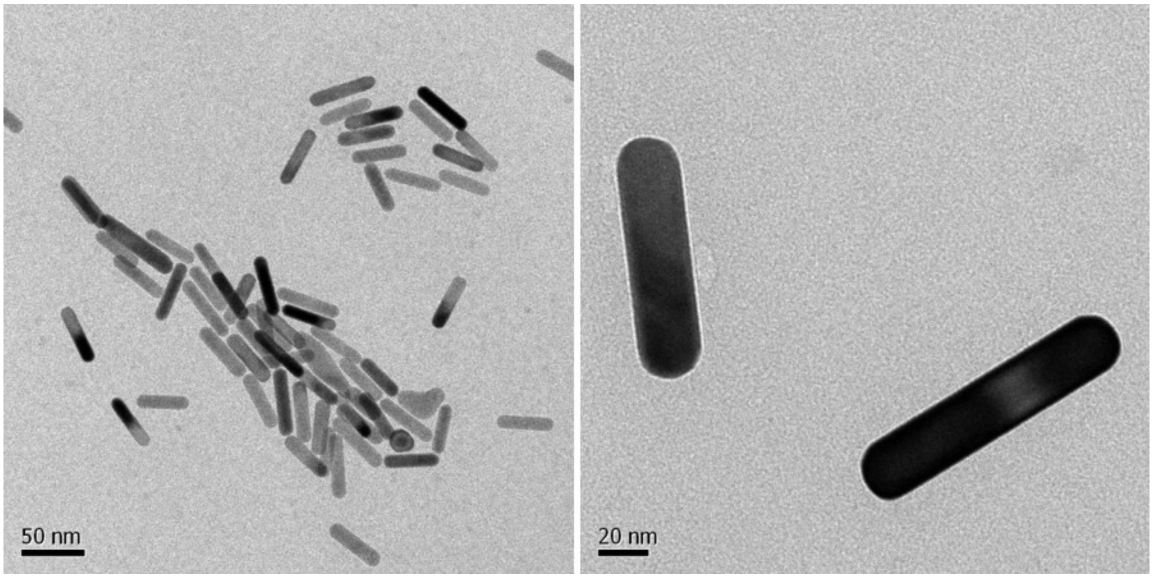

Figure S13. FETEM image of AuNRs-LA-COS solution after six cycles of laser on/off NIR laser irradiation.

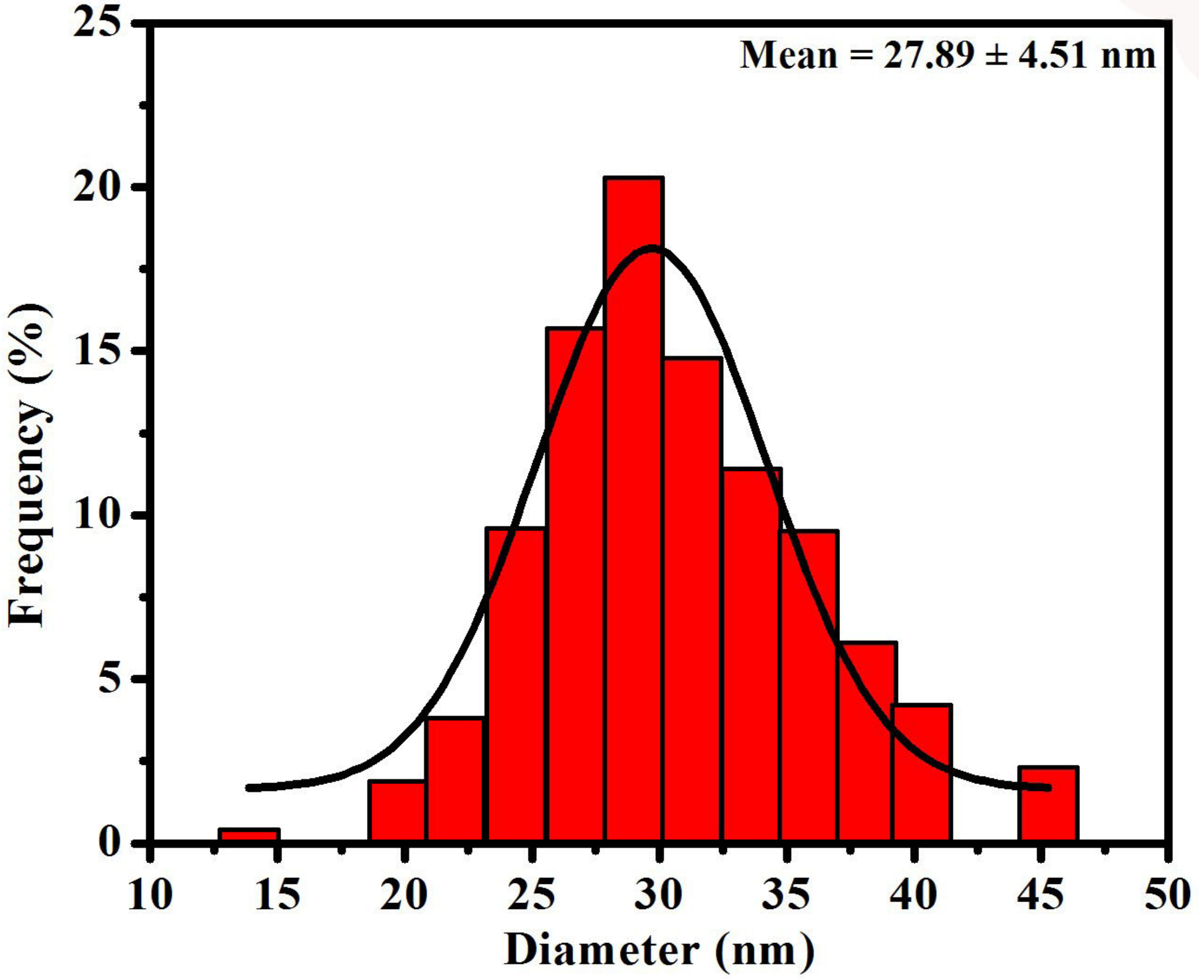

Figure S14. DLS results of AuNRs-LA-COS solution after six cycles of laser on/off NIR laser irradiation.

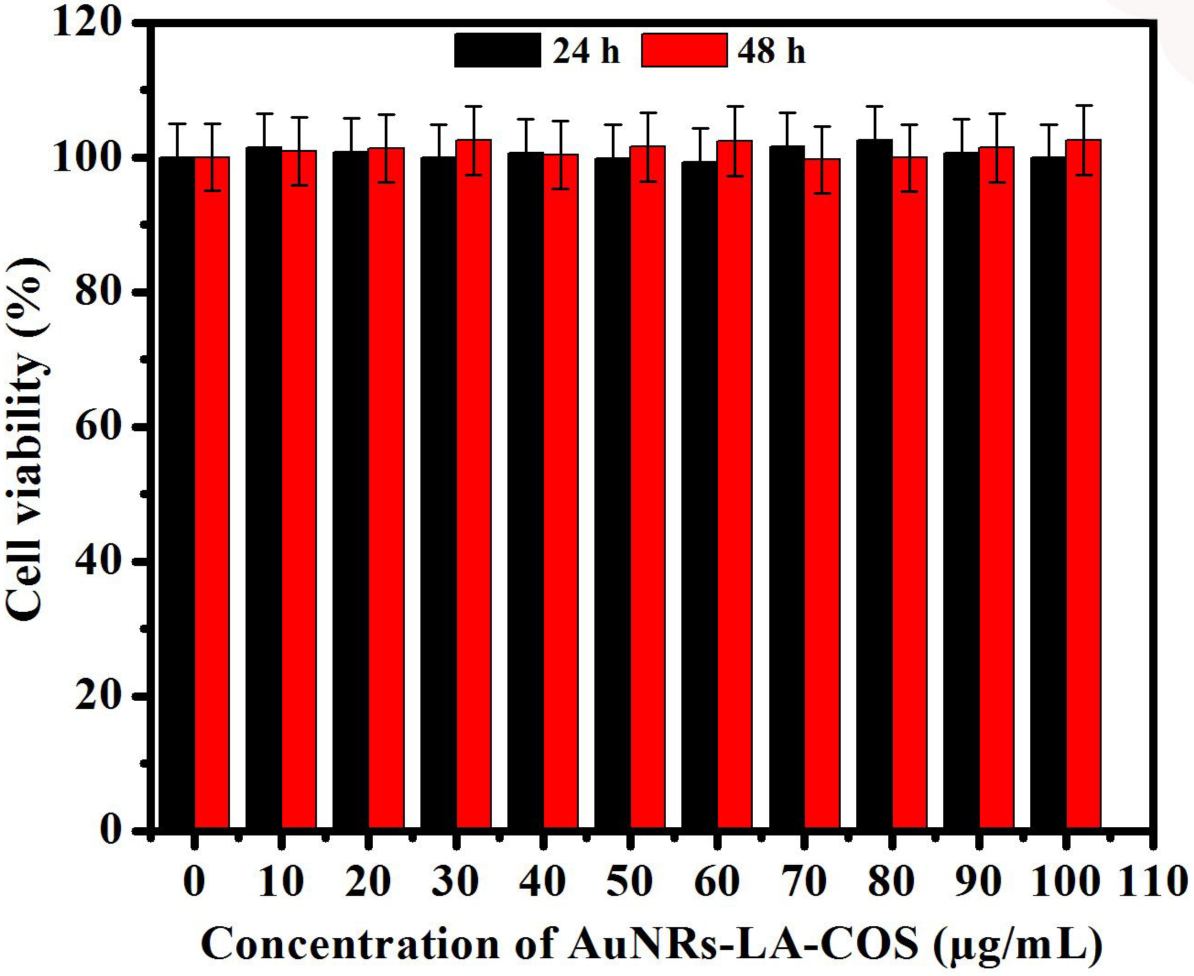

Figure S15. Biocompatibility test of AuNRs-LA-COS against HEK 293 for 24 h and 48 h. Data is expressed as mean ± SD of the three experiments.

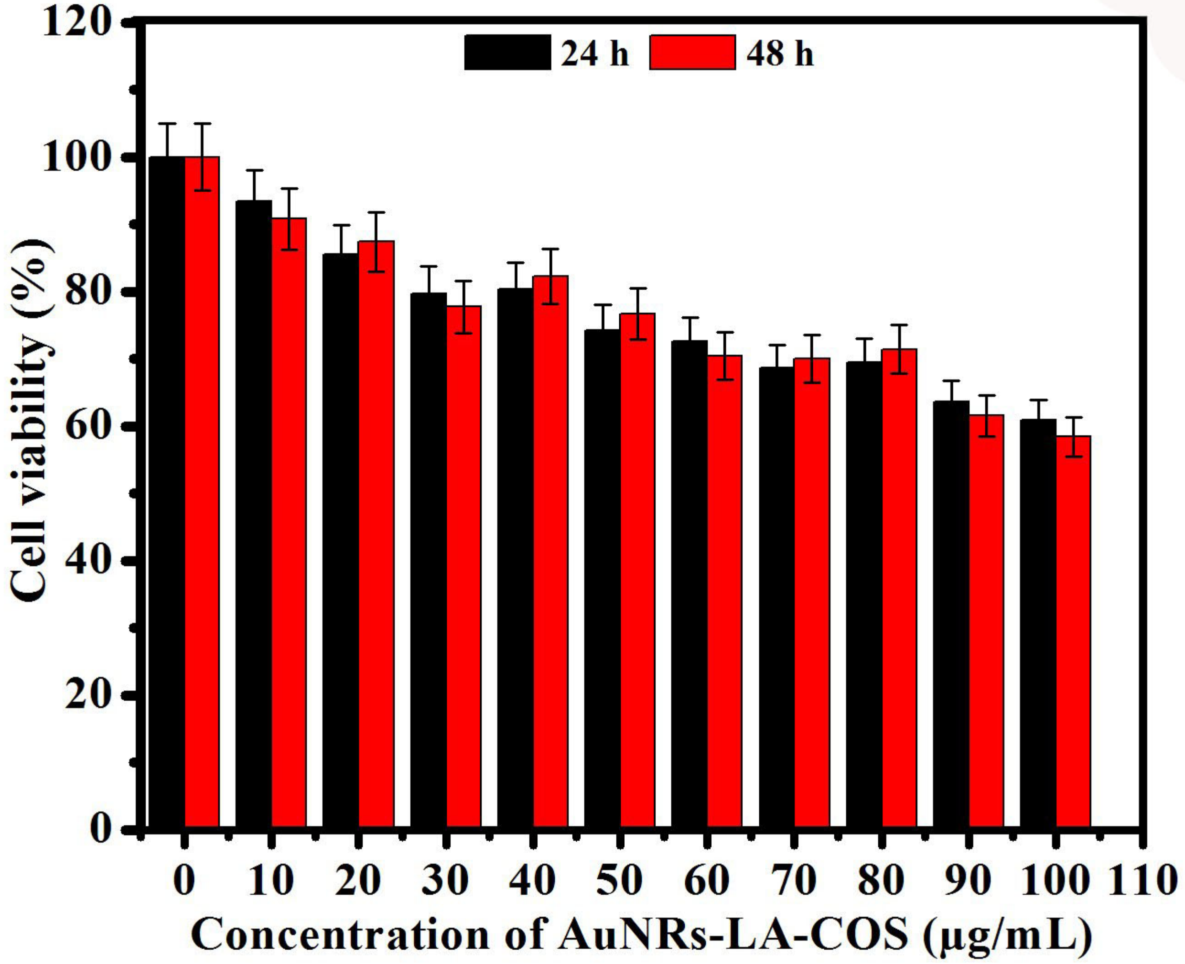

Figure S16. *In vitro* cytotoxic effect of AuNRs-LA-COS against MDA-MB-231 cells at 24 h and 48 h. Data is expressed as mean ± SD of the three experiments.

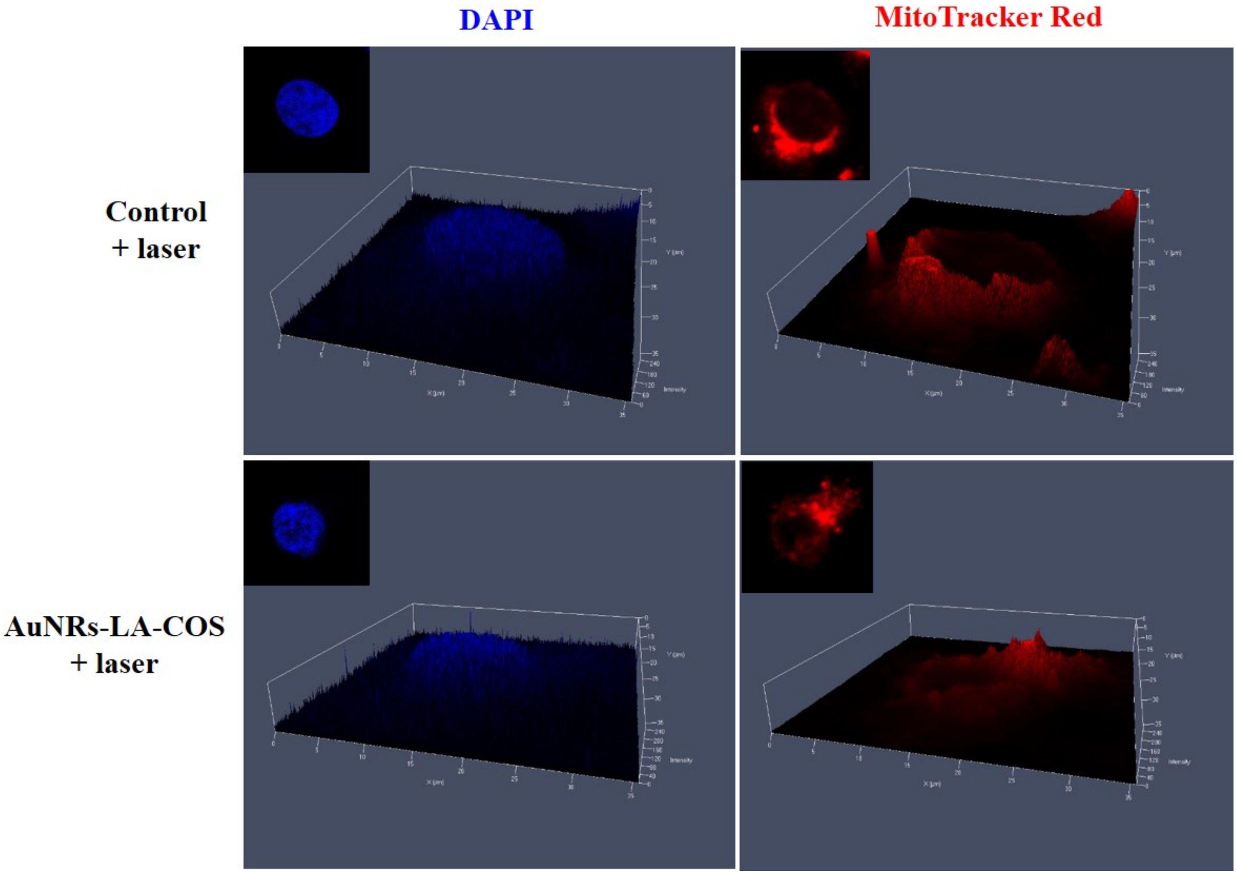

Figure S17. Confocal fluorescence microscope images stained by DAPI and MitoTracker Red. 3D confocal fluorescence microscope image of MDA-MB-231 cells treated without or with 25 μg/mL AuNRs-LA-COS and irradiated with 808 nm NIR laser at 2 W/cm^2 for 5 min.

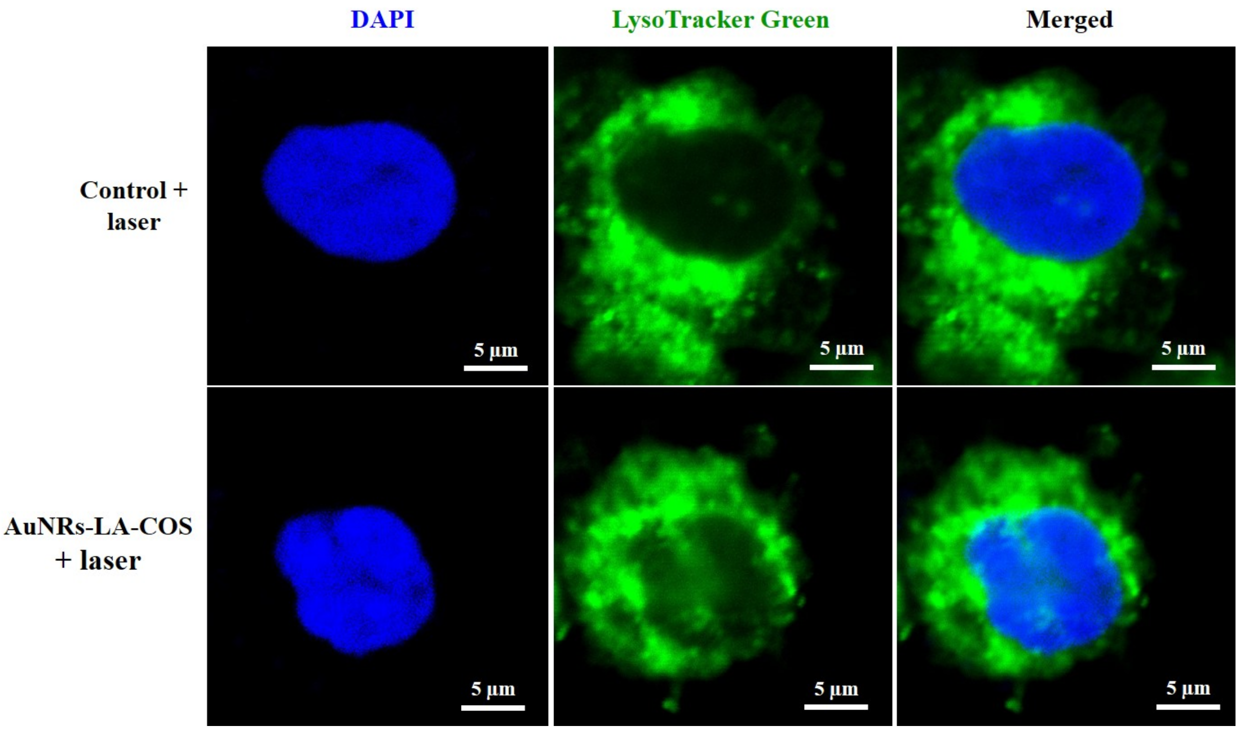

Figure S18. Confocal fluorescence microscope images stained by DAPI and LysoTracker Green. Confocal fluorescence microscope image of MDA-MB-231 cells treated without or with 25 µg/mL AuNRs-LA-COS and irradiated with 808 nm NIR laser at 2 W/cm^2 for 5 min.

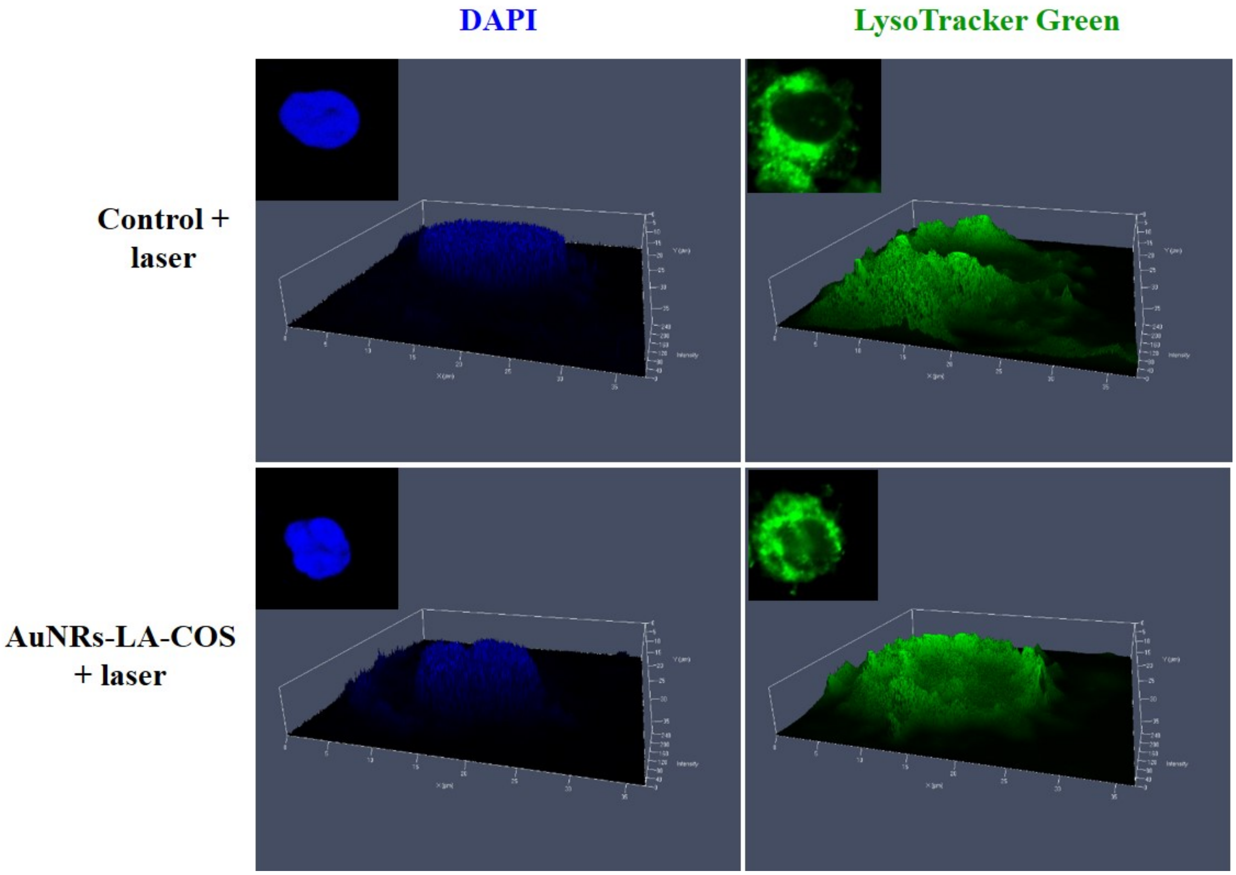

Figure S19. Confocal fluorescence microscope images stained by DAPI and LysoTracker Green. 3D Confocal fluorescence microscope image of MDA-MB-231 cells treated without or with 25 μg/mL AuNRs-LA-COS and irradiated with 808 nm NIR laser at 2 W/cm² for 5 min.

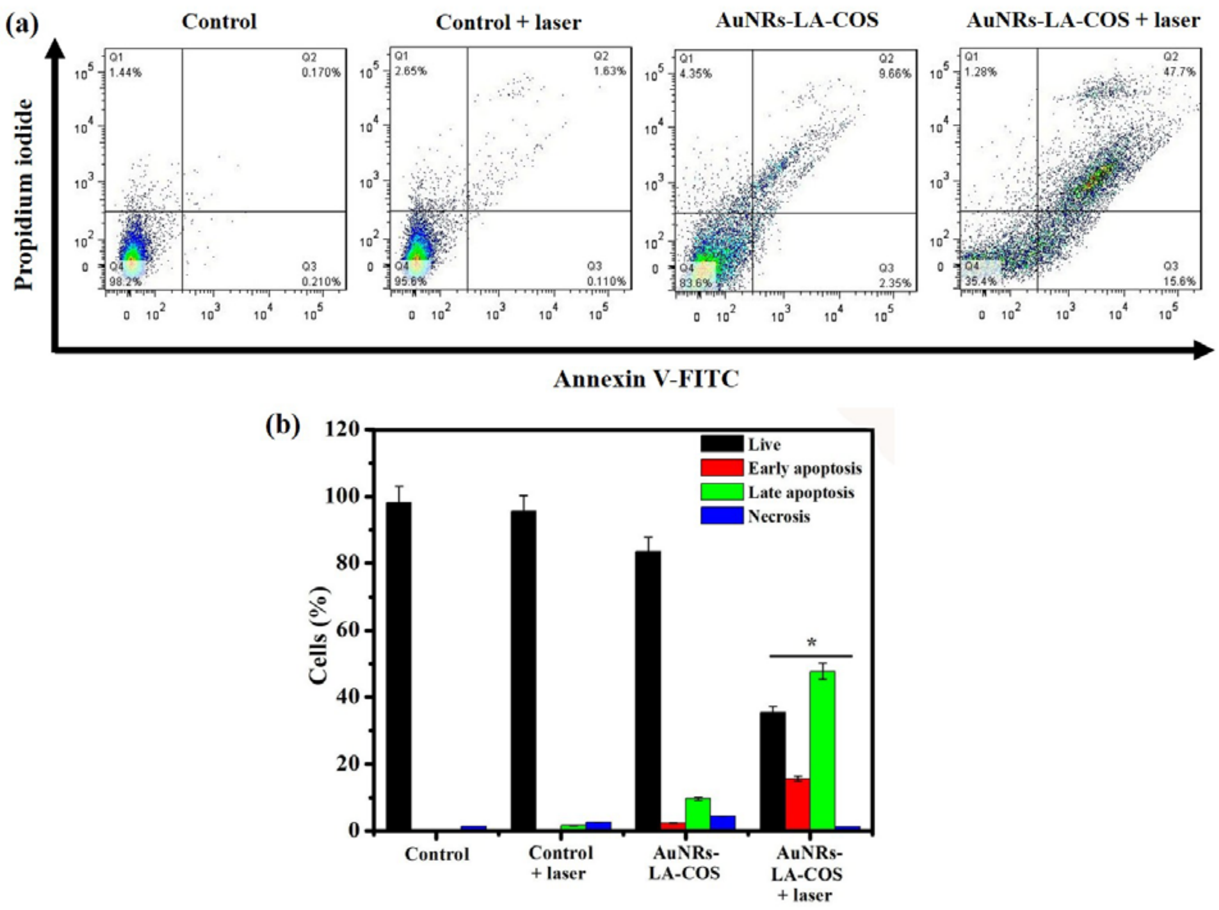

Figure S20. (a) Flow cytometry analysis of MDA-MA-231 cells incubated with or without AuNRs-LA-COS (25 µg/mL) with or without 808 nm NIR laser irradiation at 2.0 W/cm^2 for 5 min. (b) Quantified analysis of apoptotic and necrotic cells percentage according to double staining by Annexin V and PI (* significant $p < 0.05$).

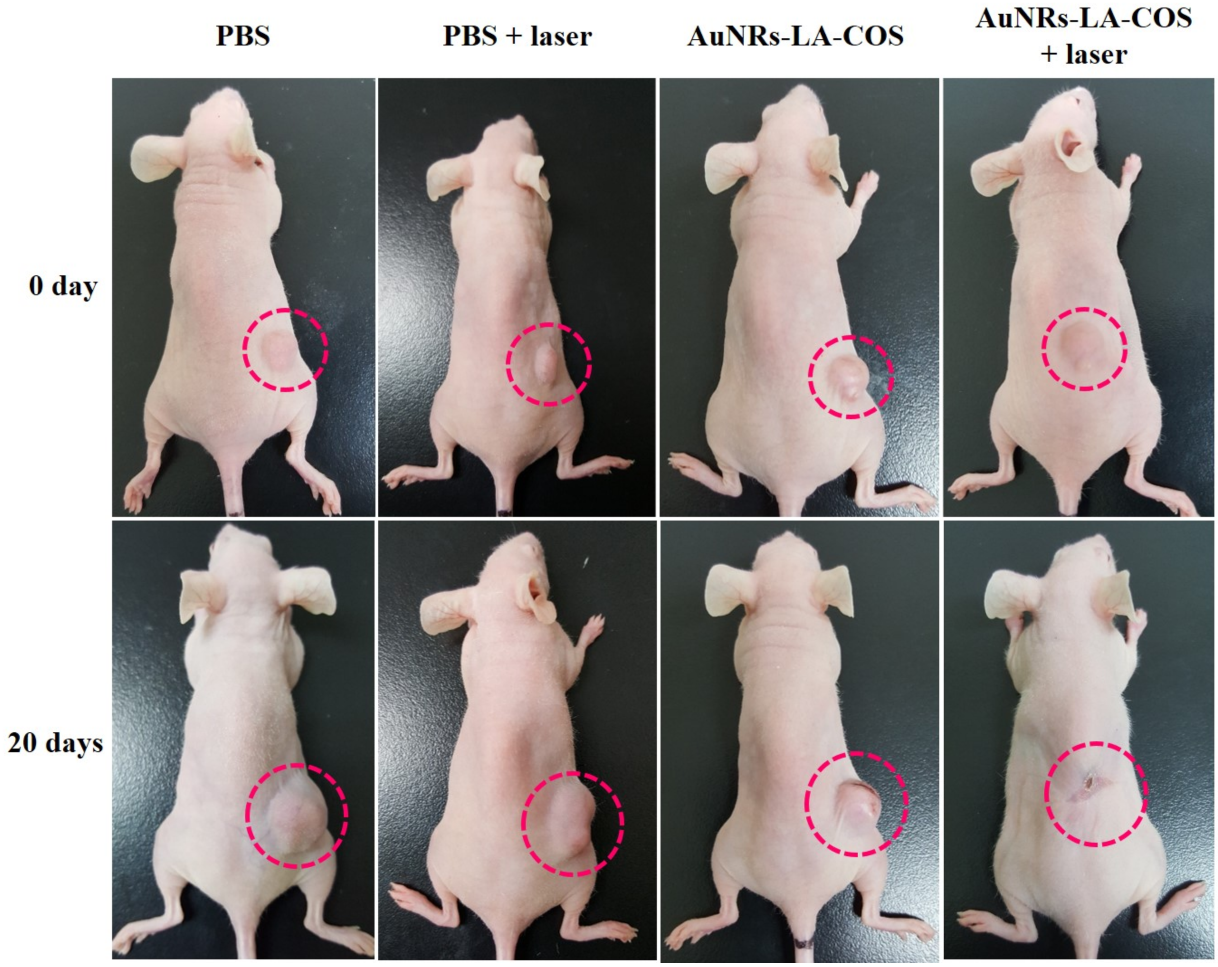

Figure S21. Photographs of mice taken before treatments (0 day) and after treatments (20 days) of AuNRs-LA-COS with an 808 nm NIR laser at 2 W/cm² for 5 min.

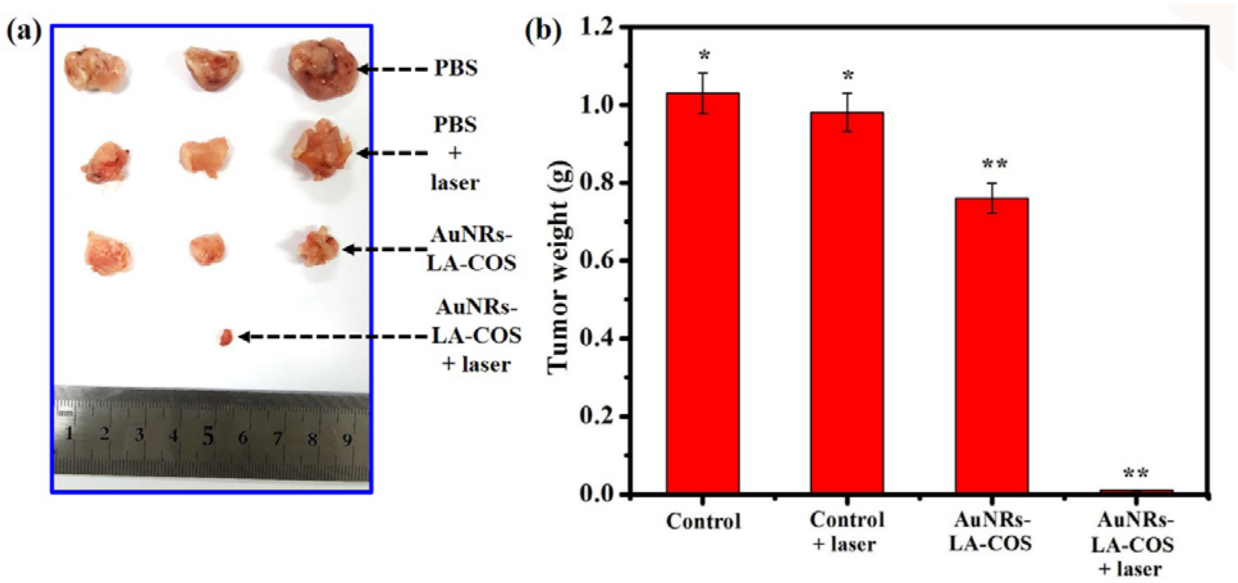

Figure S22. (a) Photographs of tumors collected from different groups of mice at the end of treatment. (b) Average weight of tumors collected from the mice at the end of PTT (* significant $p < 0.05$; ** highly significant $p < 0.01$).

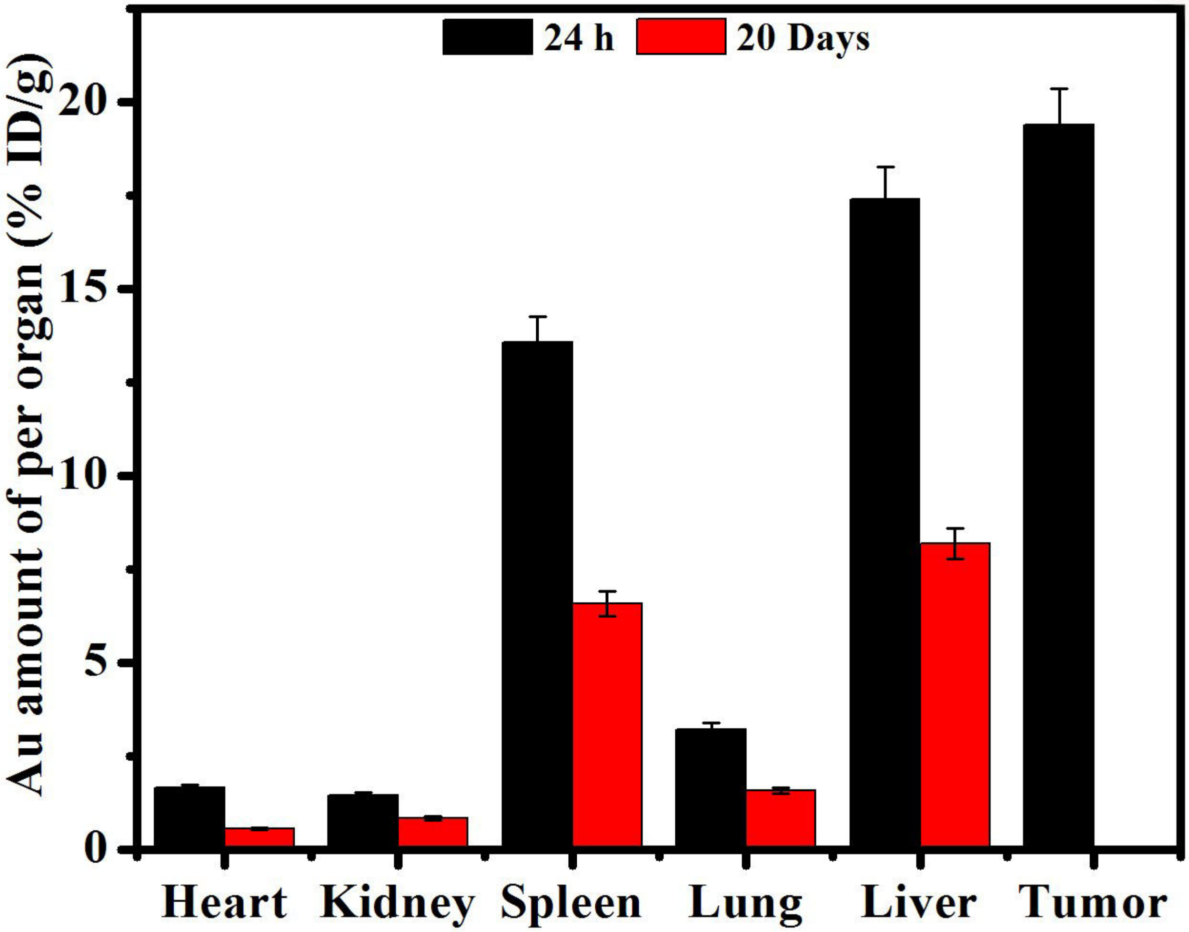

Figure S23. Biodistribution of AuNRs-LA-COS in mice at 24 h and 20 days after intratumoral injection. The gold (Au) amounts in major organs and tumor tissues were measured by ICP-MS. Data is expressed as mean ± SD of the three experiments.

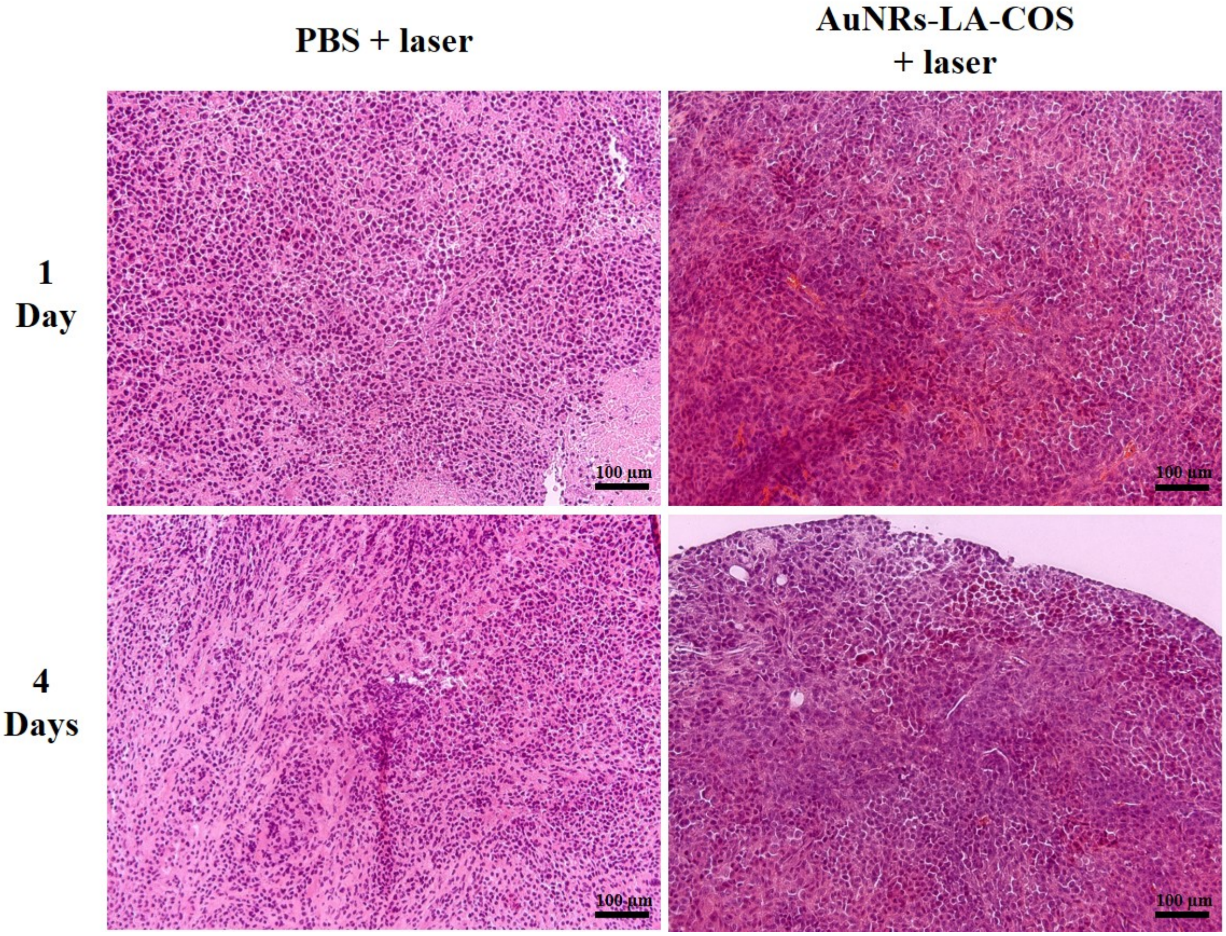

Figure 24. Histology staining of tumor tissues collected from different groups of mice after NIR laser irradiation.
